# GLP-1R activation restores Gas6-driven efferocytosis in senescent foamy macrophages to promote neural repair

**DOI:** 10.1016/j.redox.2025.103857

**Published:** 2025-09-03

**Authors:** Mingjie Xia, Chaochen Li, Yanan Zhang, Tianyi Wang, Chaoqiang Zhang, Jian Zhou, Xuexian Zhu, Hongxiang Hong, Haijun Li, Zhanyang Qian, Zhiming Cui

**Affiliations:** a Department of Spine Surgery, The Second Affiliated Hospital of Nantong University, Nantong First People's Hospital, Medical School of Nantong University, Nantong, Jiangsu, 226000, China; b Research Institute for Spine and Spinal Cord Disease of Nantong University, Nantong, Jiangsu, 226000, China; c Department of Orthopedics, The Affiliated Taizhou People's Hospital of Nanjing Medical University, Taizhou School of Clinical Medicine, Nanjing Medical University, Taizhou, 225300, China; d The Forth Clinical Medical School, Nanjing Medical University, Nanjing, 211166, China; e Key Laboratory of Neuroregeneration of Jiangsu and Ministry of Education, Nantong University, Nantong, Jiangsu, 226000, China

**Keywords:** Spinal cord injury, Macrophage senescence, Efferocytosis, Neuroinflammation, GLP-1R, Gas6

## Abstract

Spinal cord injury (SCI) is a devastating condition characterized by the accumulation of myelin debris (MD), persistent neuroinflammation, and impaired neural regeneration. Although macrophages are pivotal for MD clearance, the impact of excessive MD phagocytosis on macrophage phenotype and function remains poorly understood. Building upon our prior evidence that exendin-4 (Ex-4), a glucagon-like peptide-1 receptor (GLP-1R) agonist, mitigates microglia-driven neuroinflammation post-SCI, this study elucidates the therapeutic efficacy and underlying mechanisms of Ex-4 in alleviating macrophage senescence, restoring efferocytotic capacity, and facilitating neural repair. Employing a T10 contusive SCI model in male C57BL/6 mice, *in vivo* administration of Ex-4 was combined with macrophage-specific knockdown of growth arrest-specific 6 (Gas6) via AAV-shRNA. Complementary *in vitro* assays involved bone marrow-derived macrophages (BMDMs) challenged with MD in the presence or absence of Ex-4 or AMP-activated protein kinase (AMPK) inhibition. Cellular senescence and efferocytosis were comprehensively assessed through live-cell imaging, immunofluorescence, senescence-associated β-galactosidase staining, quantitative PCR, and western blotting. Molecular docking and dynamics simulations elucidated GLP-1R–AMPK interactions, corroborated by *in vivo* validation. Results demonstrate that MD-engulfing macrophages exhibit foam cell-like morphology and upregulated senescence markers, including increased β-galactosidase activity and senescence-associated secretory phenotype, concomitant with diminished efferocytosis via downregulation of the Axl receptor. Senescent macrophages were shown to exacerbate neuronal apoptosis and astrocytic scar formation in co-culture systems. Ex-4 treatment significantly attenuated macrophage senescence, restored efferocytotic function, and reduced neuronal injury and astrocyte activation, effects contingent upon AMPK/Gas6/Axl pathway activation and abrogated by Gas6 knockdown. *In vivo*, Ex-4 administration enhanced remyelination, axonal regeneration, and functional recovery, while attenuating glial scar formation following SCI. Collectively, these findings identify macrophage senescence induced by excessive MD phagocytosis as a novel pathological contributor to SCI progression and establish Ex-4 as a promising therapeutic agent that restores macrophage homeostasis and promotes neural repair via GLP-1R/AMPK/Gas6/Axl signaling.

## Graphical abstract

After SCI, macrophages engulf excessive MD and accumulate lipid droplets, transforming into senescent foam cells characterized by DNA damage, cell cycle arrest, and SASP secretion. These senescent macrophages display impaired efferocytosis, leading to inefficient clearance of apoptotic neurons, which in turn aggravates neuronal apoptosis and promotes astrocyte activation and glial scar formation—further hindering neural repair. Treatment with Ex-4, a GLP-1R agonist, activates the GLP-1R/AMPK/Gas6/Axl signaling pathway to mitigate macrophage senescence, enhance efferocytosis, and consequently attenuate neuronal loss and astrocytic gliosis, thereby facilitating functional recovery after SCI.

**Figure:**
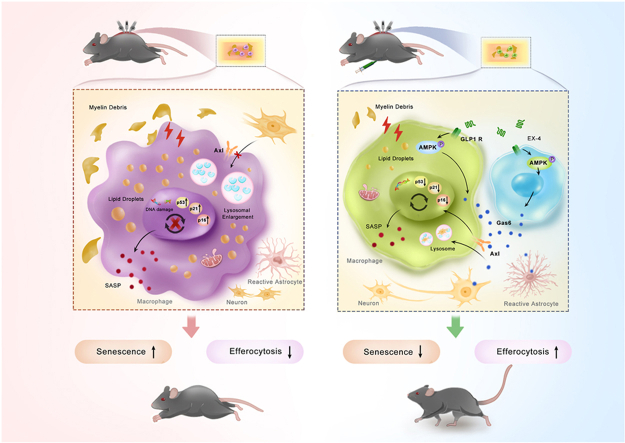

## 1 Introduction

Spinal cord injury (SCI) is a devastating medical condition characterized by disruption of the structural and functional integrity of the nervous system, which results in varying degrees of motor, sensory, and autonomic dysfunction [1,2]. The global incidence of SCI is 0.9 million (0.7–1.2) cases annually, with significant regional variability [3]. Beyond its profound medical implications, SCI imposes a substantial socioeconomic burden driven by the high costs of medical care, rehabilitation, loss of productivity, and psychological toll on both patients and their families [4]. Given the limited regenerative capacity of the central nervous system, SCI remains a critical focus of research aimed at developing effective therapeutic strategies [5]. Clinical interventions typically include conservative measures, such as edema reduction and pain management or surgical decompression of the injury site [6]. However, these approaches demonstrate limited efficacy, and the extent of neurological recovery remains unpredictable. Thus, a thorough understanding of SCI pathophysiology and identification of new therapeutic targets and their mechanisms are crucial for improving treatment strategies.

One of the key pathological SCI features is the generation of myelin debris (MD) due to mechanical disruption of axons and myelin sheaths during the initial trauma as well as secondary injury mechanisms, such as ischemia, excitotoxicity, and inflammation [7,8]. Phagocytic macrophages play a pivotal role in clearing MD and facilitating tissue repair after SCI [9]. These macrophages primarily originate from peripheral monocytes recruited to the injury site via the disrupted blood-spinal cord barrier in response to chemokines, such as monocyte chemoattractant protein-1 [10,11]. Concentrated at the injury epicenter and its periphery, macrophages are strategically positioned to interact with MD [12]. While macrophage-mediated MD phagocytosis is generally considered beneficial for injury repair, emerging evidence suggests that excessive phagocytosis can impair macrophage function [13]. When macrophages engulf large amounts of lipid-rich MD, they undergo foam cell formation characterized by intracellular accumulation of lipid inclusions [14]. These foam cells have impaired debris clearance and increase inflammation by releasing pro-inflammatory cytokines, such as tumor necrosis factor-alpha (TNF-α) and interleukin-1 beta (IL-1β) [15]. The chronic inflammatory environment exacerbates secondary injury mechanisms, contributes to glial scar formation, and hinders axonal regeneration and remyelination [16].

Efferocytosis is the process by which macrophages engulf and clear apoptotic cells and is crucial for resolving inflammation and promoting tissue repair [17,18]. During efferocytosis, macrophages recognize apoptotic cells via “eat-me” signals, such as phosphatidylserine exposure, which are detected by receptors like MerTK, Axl, and integrins [19,20]. Engulfed apoptotic cells are degraded in lysosomes, preventing the release of pro-inflammatory intracellular contents and promoting an anti-inflammatory environment [21,22]. However, impaired efferocytosis can lead to chronic inflammation and delay tissue repair [23]. Although macrophage efferocytosis has been studied extensively in other disease models such as nonalcoholic steatohepatitis, inflammatory bowel disease, and rheumatoid arthritis [[24], [25], [26]], its specific role and mechanisms in SCI remain poorly understood. Notably, studies in atherosclerosis have demonstrated that foam cells formed by macrophages exhibit impaired efferocytosis, which contributes to chronic inflammation and tissue damage [27]. It remains unclear whether efferocytosis impairment occurs in foam cells generated via excessive MD phagocytosis after SCI.

Macrophage senescence is characterized by an age-related decline in cellular function and exhibits features [28], such as telomere shortening, DNA damage accumulation, mitochondrial dysfunction, and pro-inflammatory cytokine secretion, collectively form the senescence-associated secretory phenotype (SASP) [29,30], which impairs macrophage proliferation, phagocytic function, and inflammation resolution [31]. Recent studies have highlighted that macrophages are not only essential for debris clearance but are also highly sensitive to environmental stressors within injured neural tissue [32]. In various neurological injury models, including traumatic brain injury, and ischemic stroke, macrophages exposed to persistent inflammatory cues or lipid overload exhibit hallmarks of cellular senescence, such as increased expression of senescence-associated β-galactosidase and inflammatory mediators [33]. Accumulation of MD and other lipid-rich material can exacerbate this process by promoting oxidative stress and lysosomal dysfunction within macrophages, ultimately impairing their efferocytotic capacity and contributing to sustained neuroinflammation. These observations provide a mechanistic rationale for investigating whether macrophage senescence occurs in response to excessive MD phagocytosis, thereby impairing efferocytosis, promoting astrocytic scar formation, and inhibiting axonal regeneration after SCI.

Glucagon-like peptide-1 receptor (GLP-1R) agonists, such as Exendin-4 (Ex-4), have been shown to exert neuroprotective effects in central nervous system injury [34]. Our previous work demonstrated that Ex-4 alleviates microglia-mediated neuroinflammation and promotes functional recovery following SCI, suggesting that GLP-1R activation may broadly regulate neuroimmune responses beyond microglia [35]. Mechanistically, GLP-1R signalling is closely linked to AMP-activated protein kinase (AMPK), a central regulator of cellular energy metabolism, senescence, and stress adaptation, which has been reported to restore macrophage homeostasis and suppress inflammatory phenotypes [36]. In parallel, the growth arrest-specific 6 (Gas6)/Axl axis plays a pivotal role in efferocytosis and in limiting cellular senescence, thereby preventing the accumulation of dysfunctional macrophages and sustained inflammation [37]. Based on these insights, we hypothesized that GLP-1R activation by Ex-4 could attenuate macrophage senescence and restore efferocytotic capacity after SCI through the AMPK/Gas6/Axl pathway, ultimately facilitating neural repair.

This study reports for the first time that macrophages undergoing excessive MD phagocytosis exhibit senescence after SCI, resulting in impaired efferocytosis. Notably, senescent macrophages also inhibited axonal regeneration and promoted astrocytic scar formation, further exacerbating the hostile microenvironment for neural repair. Specifically, administration of the GLP-1R agonist Ex-4 in SCI mice promoted AMPK phosphorylation and subsequently activated the Gas6/Axl signaling pathway. This cascade suppressed macrophage senescence, restored efferocytosis capacity, attenuated astrocyte reactivity, supported axonal outgrowth, and ultimately facilitated neurological recovery. These findings highlight the regulatory role of GLP-1R activation in modulating macrophage senescence and its downstream effects on efferocytosis and glial-neuronal interactions post-SCI, providing a novel pathophysiological basis and a promising therapeutic direction for promoting neurological recovery after SCI.

## 2 Materials and methods

### 2.1 Animals

All experiments involving animals were conducted according to the ethical policies and procedures approved by the Ethics Committee of Nantong First People's Hospital in accordance with the Basel Declaration (Approval No. S20230727-007, Date: July 27, 2023). The Ethics Committee of Nantong First People's Hospital acts on the International Council for Laboratory Animal Science (ICLAS). All animal treatments, behavioral assessments, histological analyses, and data quantifications were performed by investigators blinded to the group assignments. Sample identities were coded during data collection and analysis to ensure unbiased evaluation.

**Fig. S1:**
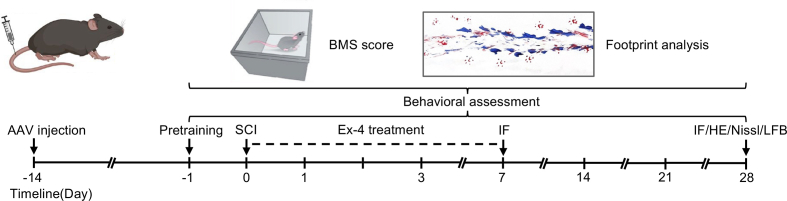
Schematic diagram of animal experimental design and implementation Illustration of the overall timeline and procedures used in the *in vivo* experiments, including SCI induction, pharmacological treatments, and sample collection.

Adult male C57BL/6 mice (20–25 g) were purchased from Huachuang Sino Pharma Tech Co. Ltd. (license no. SCXK 2020-0009, Taizhou, China). Mice were housed in specific pathogen-free facilities under controlled conditions: temperature 20–26 °C, relative humidity 40–70 %, and a 12-h light/dark cycle. Sterilized rodent chow and autoclaved water were provided to meet nutritional requirements, while regular monitoring and disinfection of cages were performed to ensure animal experiment stability. The schematic diagram of animal experimental design and implementation is provided in Fig. S1.

### 2.2 Mouse SCI model establishment

A standardized mouse SCI model was established as previously described [38]. Briefly, adult mice were anesthetized with a ketamine (100 mg/kg) and xylazine (20 mg/kg) mixture to achieve surgical anesthesia and analgesia. After shaving and disinfecting the surgical area, a midline skin incision was made to expose the dorsal vertebral column, and the paraspinal muscles were gently retracted to reveal the T10 vertebra. A laminectomy was then performed at T10 to expose the dorsal surface of the spinal cord without causing mechanical damage. A controlled contusion injury was induced using a pneumatic-electronic precision impactor (68099Ⅱ, RWD, Shenzhen, China), ensuring consistency and reproducibility. The incision was closed in layers, and mice were placed on a warming pad for postoperative recovery. Successful SCI induction was confirmed by the appearance of a central spinal cord hematoma, a tail-flick response during impact, and complete hind limb paralysis. To aid urination, manual bladder expression was performed once daily for one week until spontaneous bladder function returned. Mice in the Sham group underwent identical surgical procedures including anesthesia and T10 laminectomy but without contusive SCI.

### 2.3 *Single-cell* RNA sequencing (scRNA-seq) *bioinformatics data acquisition*

The scRNA-seq data of both normal and injured spinal cord samples at 1, 3, and 7 days post-injury (dpi) in wild-type mice were sourced from the GEO database (accession number: GSE162610). Senescence-related genes (SRGs) were identified through a search of the Mouse Genome Informatics (MGI) database using the term "senescence." In addition, efferocytosis-related genes (ERGs) were selected by querying both the GeneCards and MGI databases with the keyword "efferocytosis." The complete lists of these genes are provided in Table S1.

### 2.4 Single-cell subtype Classification and Annotation in SCI

Following established protocols using the Seurat4 package, we normalized, scaled, transformed, integrated, and corrected for cell cycle effects on the raw sequencing data. Dimensional reduction, neighbor identification, and clustering analyses were subsequently performed using Seurat4 to explore the underlying cellular structure. Marker genes for each cluster were identified using the *FindAllMarkers* function, with cluster-specific markers calculated relative to the entire dataset. Pairwise marker comparisons were conducted using the *FindMarkers* function to identify differences between clusters representing subtypes within the same broad cell identity. Cell clusters were annotated based on differentially expressed genes (DEGs) and known markers from existing literature. The proportions of various cell types were visualized through Uniform Manifold Approximation and Projection (UMAP). To further investigate cell type functions, enrichment analysis of DEGs was performed using the *clusterProfiler* package, focusing on Gene Ontology (GO) biological processes and Kyoto Encyclopedia of Genes and Genomes (KEGG) pathways, with statistical significance defined by an adjusted P-value of <0.05.

### 2.5 Scoring of SRG and ERG activity across different cell types

To assess the activity of SRGs and ERGs across various cell types, we employed five distinct scoring methods: AUCell, singscore, UCell, ssGSEA, and AddModuleScore. These approaches were designed to capture the intrinsic relationships between cell types, states, and active biological processes. Cells exhibiting high expression levels of SRGs or ERGs were assigned elevated scores. These scores were then visualized using UMAP, with color-coding to highlight clusters where SRG or ERG activity was most pronounced.

### 2.6 Adeno-associated virus (AAV) vector packaging and injection

A short hairpin RNA (shRNA) sequence targeting *Gas6* was cloned into a vector under the control of the F4/80 promoter, which specifically drives expression in macrophages. The construct was then packaged into an adeno-associated virus vector (AAV-shGas6; GeneChem, Shanghai, China) for *Gas6* knockdown. A scrambled shRNA sequence was used as a negative control (AAV-NC). The target interference sequence for *Gas6* was 5′-GCTCAGTGACTATGCTTAA-3′. For systemic AAV delivery, mice were restrained and their tails disinfected with 70 % ethanol. A total volume of 200 μL viral solution (1.33 × 10^12^ vg/mL) was injected into the lateral tail vein using a 30-gauge syringe, allowing for macrophage-specific transduction via the F4/80 promoter.

### 2.7 In vivo Ex-4 treatment

Ex-4 (HY-13443, MedChemExpress) was dissolved in sterile 0.9 % saline to prepare a stock solution (1 mg/mL) and stored at −20 °C in aliquots to avoid repeated freeze–thaw cycles. For *in vivo* experiments, Ex-4 was freshly diluted in saline to a working concentration prior to use. In mice subjected to SCI, Ex-4 was administered intraperitoneally at a dose of 20 μg/kg per mouse using a sterile insulin syringe. Injections were performed once daily at the same time each day for seven consecutive days post-injury to ensure dosing consistency and minimize circadian-related variability. The dosage and administration frequency were selected based on previously published studies of Ex-4 in models of central nervous system injury [39], as well as our preliminary dose-finding experiments, which confirmed efficacy in reducing neuroinflammation without observable adverse effects.

### 2.8 Oil Red O (ORO) staining

Frozen spinal cord sections were equilibrated to room temperature and fixed in 4 % paraformaldehyde for 15 min. The ORO working solution was prepared by mixing six parts of saturated ORO solution (Servicebio, Wuhan, China) with four parts of distilled water, followed by incubation at 4 °C overnight. Sections were stained with the ORO working solution for 10 min in the dark to prevent photobleaching. Background differentiation was performed by sequential immersion in two 60 % isopropanol baths (3 s and 5 s, respectively), followed by a brief rinse (10 s each). Nuclei were counterstained with hematoxylin for 5 min. Finally, sections were mounted using neutral balsam and visualized under a microscope (Nikon, Tokyo, Japan) for image acquisition and subsequent analysis.

### 2.9 Hematoxylin and Eosin (HE), Luxol Fast Blue (LFB), and Nissl staining

Paraffin-embedded spinal cord sections were deparaffinized in xylene and rehydrated through a descending ethanol gradient (100 %, 95 %, 80 %, and 70 %), followed by rinsing in distilled water. For HE staining, sections were stained with hematoxylin for 10 min to visualize nuclei, differentiated using acid alcohol, and then stained with eosin for 3 min to label the cytoplasm and extracellular components. After rinsing in distilled water, sections were dehydrated through ascending ethanol concentrations (70 %, 80 %, 95 %, and 100 %), cleared in xylene, and mounted using neutral balsam. For Nissl staining, sections were stained with toluidine blue for 15 min at room temperature to label Nissl bodies and neuronal structures. Nissl bodies appeared as dark blue granules within the cytoplasm. For LFB staining, Myelin Staining Solution A (Servicebio) was preheated to 60 °C for 30 min. Sections were then immersed in the solution and stained for 1 h. Differentiation was performed by briefly immersing the sections in warm Myelin Staining Solution B for 2 s, followed by Myelin Staining Solution C for 15 s. For counterstaining, sections were dried at 65 °C for 30 min, cooled, immersed in 95 % ethanol, and then counterstained with eosin. All stained sections were subsequently dehydrated through graded ethanol, cleared in xylene, mounted with neutral balsam, and examined under a microscope (Nikon) for imaging and histological analysis.

### 2.10 Immunofluorescence (IF) staining

For IF staining, cultured cells were fixed with 4 % paraformaldehyde for 15 min at room temperature. Paraffin-embedded tissue sections were deparaffinized, rehydrated through a graded ethanol series, and subjected to heat-induced antigen retrieval using sodium citrate buffer (Beyotime, Nanjing, China). Endogenous peroxidase activity was quenched by incubating the sections with a peroxidase blocking solution (Beyotime) for 30 min. Non-specific binding sites were blocked by treating the samples with a blocking buffer (Beyotime) for 1 h at room temperature. Subsequently, samples were incubated overnight at 4 °C with primary antibodies diluted in blocking buffer. After washing, samples were incubated with appropriate fluorophore-conjugated secondary antibodies for 1 h at room temperature in the dark. Nuclear counterstaining was performed using 4′,6-diamidino-2-phenylindole (DAPI) for 10 min. Finally, samples were mounted using an anti-fade mounting medium and visualized under a fluorescence microscope (Nikon) for image acquisition and analysis.

### 2.11 MD preparation

C57BL/6 mice (8–12 weeks old) were euthanized by cervical dislocation, followed by immersion in 75 % ethanol for 15 min and decapitation. Brains were rapidly removed using sterile surgical scissors and forceps, and transferred to a pre-chilled 10-cm culture dish containing 10 mL of ice-cold Tris·Cl buffer (prepared by mixing 480 mL ddH_2_O, 10 mL 1 M Tris·Cl, and 10 mL 0.5 M Na_2_EDTA). After rinsing to remove surface impurities, the brain tissue was transferred into a 50-mL sterile centrifuge tube.

Next, 0.32 M sucrose solution was added to the tube to a final volume of 25 mL. The brain tissue was homogenized for 2 min and kept on ice. For density-gradient centrifugation, 3 mL of 0.83 M sucrose solution was added to eight ultracentrifuge tubes. The homogenate was gently layered over the sucrose solution, taking care not to mix the layers. Tubes were balanced on an analytical scale and ultracentrifuged at 100,000×*g* for 45 min at 4 °C. After centrifugation, the white myelin debris band at the interface was carefully collected, resuspended in 2 mL Tris·Cl buffer, and homogenized. The suspension was then distributed into four ultracentrifuge tubes (5 mL per tube) and centrifuged again at 100,000×*g* for 45 min. The resulting pellets were resuspended in 2 mL Tris·Cl buffer and pooled into two tubes. A final centrifugation was performed at 22,000×*g* for 10 min at 4 °C. The resulting myelin pellet was weighed, resuspended in phosphate-buffered saline (PBS) to a final concentration of 100 mg/mL, and stored at −80 °C for up to six months.

### 2.12 Extraction, culture and treatment of mouse bone marrow-derived macrophages (BMDMs)

Following euthanasia under deep anesthesia, 6–8-week-old mice were immersed in 75 % ethanol, and their femurs and tibias were carefully dissected. Muscles and connective tissues were completely removed. The bones were disinfected again with 75 % ethanol and transferred into a sterile biosafety cabinet. Both ends of the femurs and tibias were cut, and bone marrow was flushed out by injecting 5 mL of Dulbecco's Modified Eagle Medium (DMEM; KeyGEN, Nanjing, China) into the marrow cavity using a syringe. The cell suspension was passed through a sterile cell strainer to remove debris, and the flushing procedure was repeated twice to ensure maximal cell recovery. The collected bone marrow cells were treated with red blood cell lysis buffer (Beyotime) for 3 min at room temperature to remove erythrocytes. After centrifugation at 1300 rpm for 5 min, the supernatant was discarded, and the cell pellet was resuspended in DMEM supplemented with 5 % fetal bovine serum (FBS; Gibco, Grand Island, NY, USA) and 30 ng/mL macrophage colony-stimulating factor (M-CSF; HY-P70263A, MedChemExpress, USA). Cells were cultured in this medium for 3 days, followed by a medium change. Differentiation into BMDMs was achieved by continuous culture for an additional 5–7 days. For functional assays, BMDMs were pretreated with either the p53-specific inhibitor Pifithrin-β (PFT-β, 10 μM for 24 h; HY-16702A, MedChemExpress), Exendin-4 (Ex-4, 100, 200, or 400 nM for 24 h; HY-13443, MedChemExpress), compound 5d (1 μM for 24 h, HY-101116, MedChemExpress), recombinant Mouse Gas6 (rGas6, 500 ng/mL for 2h; 986-GS-025/CF, R&D Systems, Minneapolis, MN, USA) or BML-275 (1 μM for 24 h; HY-13418A, MedChemExpress), followed by stimulation with MD (1 mg/mL) for 12 h.

### 2.13 Senescence-associated β-galactosidase (SA-β-gal) staining

SA-β-gal staining was performed using a β-Galactosidase Staining Kit (C0602, Beyotime, China) following the manufacturer's instructions. Briefly, BMDMs cultured in 6-well plates were washed once with PBS and fixed with 1 mL of fixative solution at room temperature for 15 min. After fixation, the cells were rinsed three times with PBS and then incubated with 1 mL of freshly prepared SA-β-gal staining solution at 37 °C in a dry incubator (no CO_2_) overnight. On the following day, stained cells were examined under a light microscope (Nikon) for the presence of characteristic blue-stained granules indicating senescence.

### 2.14 Primary astrocyte and neuron isolation

Primary astrocytes were isolated from the cerebral cortices of postnatal day 1–3 C57BL/6J mice. Cortical tissues were dissected on ice and minced into ∼2 × 2 mm fragments. After centrifugation at 800 rpm for 3 min, the tissues were enzymatically digested with 0.25 % trypsin (Gibco, USA) at 37 °C for 5 min. The digestion was terminated by adding DMEM (Gibco) supplemented with 10 % FBS (Gibco). The tissue suspension was gently triturated using a flame-polished Pasteur pipette, filtered through a 70 μm cell strainer, and centrifuged at 1300 rpm for 5 min. The resulting pellet was resuspended in DMEM containing 10 % FBS and seeded into poly-d-lysine (PDL; 50 μg/mL; Sigma-Aldrich, St. Louis, MO, USA)-coated T25 flasks. Cells were maintained at 37 °C in a humidified incubator with 5 % CO_2_. The culture medium was replaced after 24 h and subsequently every three days. After 14–20 days, microglia were removed by shaking the flasks at 200 rpm for 3–4 h at 37 °C. To further purify astrocytes, an additional shaking step (12 h at 200 rpm) was performed, followed by trypsinization for downstream applications.

Primary cortical neurons were isolated from embryonic day 16–18C57BL/6J mouse embryos. Following dissection on ice, the cerebral cortices were minced and digested with 2 mg/mL papain (Sigma-Aldrich) in DMEM at 37 °C for 20–30 min, with gentle agitation every 5 min. The enzymatic reaction was terminated using DMEM containing 2 % horse serum (Gibco). The suspension was gently triturated on ice, filtered through a 70 μm cell strainer, and centrifuged at 1000 rpm for 5 min. The cell pellet was resuspended in DMEM with 10 % FBS and plated onto PDL-coated 6-well plates at a density of 7 × 10^5^ cells/well. After 4 h of attachment, the culture medium was replaced with Neurobasal medium (Gibco) supplemented with 1 × GlutaMAX (Gibco) and 2 % B27 supplement (Gibco). Neurons were maintained at 37 °C in a 5 % CO_2_ incubator, with medium changes every two days.

### 2.15 Preparation and labeling of apoptotic neurons

Primary cortical neurons were isolated from embryonic day 16–18 mice as previously described. After 7 days of *in vitro* culture, apoptosis was induced by treating the neurons with 1 μM staurosporine (Sigma-Aldrich) at 37 °C for 4 h. Following treatment, the cells were washed twice with PBS and resuspended in serum-free DMEM. The apoptotic neurons were then co-cultured with BMDMs for assessment of efferocytosis by live-cell imaging. For IF staining, apoptotic neurons were labeled with 5 μM carboxyfluorescein diacetate succinimidyl ester (CFSE; MedChemExpress) by incubation at 37 °C for 15 min in the dark. After labeling, cells were washed three times with PBS and subsequently co-cultured with BMDMs to visualize the digestion of apoptotic neurons within macrophage lysosomes.

### 2.16 Indirect co-culture of BMDMs with primary astrocytes and neurons

Culture supernatants from treated BMDMs were collected, centrifuged at 300×*g* for 5 min, and filtered through a 0.22 μm membrane to obtain macrophage-conditioned medium (MCM). The MCM was then applied to primary astrocytes or neurons, which were incubated for 24 h. Subsequent assays were conducted to evaluate astrocytic scar formation, neuronal axonal growth, and neuronal apoptosis.

### 2.17 Flow cytometry (FCM)

Neuronal apoptosis was assessed using an Annexin V-FITC/PI Apoptosis Detection Kit (YFXCA03, YIFEIXUE BioTech, Nanjing, China). Primary neurons were harvested by trypsin digestion (Gibco) and centrifuged at 1000 rpm for 5 min. After washing with PBS, cells were resuspended in 100 μL of 1 × Binding Buffer and incubated with 5 μL Annexin V-FITC and 5 μL PI for 15 min at room temperature in the dark. Samples were subsequently analyzed on a flow cytometer (FACSVerse 8, BD Biosciences, NJ, USA).

### 2.18 Lentivirus (LV) transfection

BMDMs were transduced with LV-NC or LV-shGas6 (1 × 10^8^ TU/mL) in the presence of 1 × HitransG A (GeneChem) as a transduction enhancer. Cells were incubated at 37 °C with 5 % CO_2_ for 12 h. After incubation, the viral-containing medium was gently removed and replaced with complete growth medium. The cells were then cultured for an additional 60 h before subsequent experiments.

### 2.19 Panoramic imaging for label-free live cells with an optical diffraction tomography (ODT) microscope

Label-free imaging of BMDMs under different experimental conditions was performed using a live-cell super-resolution panoramic microscope (MH-Holiview, Cheng Guan Optics Technology Co., Ltd., Nantong). This system is based on an off-axis holographic ODT setup integrated into a commercial microscope (IX83, Olympus). The platform incorporates a galvo-mirror scanning mechanism, a fluorescence excitation module, and image acquisition software developed in MATLAB 2021a. These components collectively enable high spatiotemporal resolution dynamic imaging of live BMDMs and support cross-scale panoramic visualization of cellular and organelle interactions.

### 2.20 Western blot (WB)

Proteins were extracted from BMDMs using lysis buffer supplemented with protease and phosphatase inhibitors (KeyGEN) and quantified by bicinchoninic acid assay. Equal amounts of protein were denatured and separated by sodium dodecyl sulfate-polyacrylamide gel electrophoresis according to molecular weight, then transferred onto polyvinylidene fluoride membranes. Membranes were blocked with 5 % non-fat milk for 1 h at room temperature to prevent non-specific binding, followed by overnight incubation at 4 °C with primary antibodies against target proteins. After washing to remove unbound antibodies, membranes were incubated with appropriate secondary antibodies. Protein bands were visualized using an imaging system (Syngene, Cambridge, UK) and quantified with ImageJ software (NIH, Bethesda, MD, USA). Detailed information on antibodies used is listed in Table S2.

### 2.21 RNA isolation and real-time quantitative PCR (qPCR)

Total RNA was extracted from cells using TRIzol reagent (YIFEIXUE) following the manufacturer's protocol. RNA concentration was measured with a spectrophotometer, and purity was assessed by the A260/A280 ratio. Subsequently, 1 μg of RNA was reverse-transcribed into complementary DNA (cDNA) using a reverse transcription kit (YIFEIXUE). qPCR was performed in 96-well plates using SYBR Green Master Mix on a Roche LightCycler 480 system (Roche, Basel, Switzerland). Each 20 μL reaction contained cDNA template, specific primers, SYBR Green mix, and nuclease-free water. The thermal cycling conditions were: initial denaturation at 95 °C for 10 min, followed by 40 cycles of 95 °C for 15 s and 60 °C for 1 min. Relative gene expression was calculated using the comparative Ct (2^−ΔΔCt) method, normalized to β-actin. Primer sequences are listed in Table S3.

### 2.22 Co-immunoprecipitation (Co-IP)

Co-IP assays were performed to examine the interaction between GLP-1R and AMPK. Briefly, BMDMs were lysed in IP lysis buffer (20 mM Tris-HCl, pH 7.5, 150 mM NaCl, 1 % NP-40, 1 mM EDTA, 10 % glycerol) supplemented with protease and phosphatase inhibitors (KeyGEN). Cell lysates were clarified by centrifugation at 12,000×*g* for 15 min at 4 °C, and the supernatants were collected. Protein concentrations were determined using the BCA assay. For IP, 500 μg of total protein was incubated overnight at 4 °C with 2 μg of anti-GLP-1R antibody (sc-390774, Santa Cruz Biotechnology, Dallas, TX, USA) or anti-AMPKα antibody (#2532, Cell Signaling Technology, Danvers, MA, USA), with gentle rotation. Normal rabbit IgG (#2729, Cell Signaling Technology) was used as a negative control. The immune complexes were captured by incubation with 30 μL of Protein A/G agarose beads (MedChemExpress) for 2 h at 4 °C. Beads were washed five times with cold lysis buffer, and bound proteins were eluted by boiling in SDS loading buffer for 10 min. Eluted samples were subjected to SDS-PAGE and WB. Protein bands were visualized using an imaging system (Syngene).

### 2.23 Molecular docking

Protein sequences of GLP-1R (Protein Data Bank (PDB) ID: 7DUQ) and AMPK (PDB ID: 6E4U) were retrieved from the PDB. Structures were predicted using AlphaFold3, with low-confidence terminal regions removed. Protein-protein docking was performed using the HDOCK server, which utilizes a hybrid docking strategy to generate potential complex models. Docking results were analyzed and visualized using PyMOL (version 3.0.3). Docking scores were calculated via knowledge-based iterative scoring functions (ITScorePP or ITScorePR), where more negative scores indicate higher binding affinity and more reliable docking models.

### 2.24 Molecular dynamics simulation of the GLP-1R–AMPK complex

Molecular dynamics simulations were conducted using AMBER24 software (University of California, San Francisco, USA) [40] to investigate the dynamics of the GLP-1R–AMPK complex. The ff19SB force field [41,42] and Optimal Point Charge water model [43] were applied. The complex was solvated in a cubic water box with a 10 Å buffer. Electrostatic and van der Waals interactions were truncated at 1.0 nm, with long-range electrostatics treated by the particle mesh Ewald method. Simulations were performed at 300 K and 1 bar using a 2 fs integration time step. After energy minimization [44], the system was equilibrated under constant volume for 200 ps and constant pressure for 100 ps. Temperature coupling was maintained via the velocity-rescale thermostat, and pressure was controlled using the Parrinello–Rahman barostat. A 100 ns production run was then carried out. Trajectory analyses including root-mean-square deviation (RMSD), root-mean-square fluctuation (RMSF), radius of gyration (Rg), solvent-accessible surface area (SASA), and hydrogen bond count were performed using AMBER's Cpptraj and custom Python scripts.

### 2.25 Behavioral evaluation

Hindlimb locomotor function in SCI mice was assessed using the Basso Mouse Scale (BMS) [45]. Mice were placed in an open field, and hindlimb movements were observed and scored from 0 (complete paralysis) to 9 (normal locomotion), based on parameters such as joint movement, stepping ability, coordination, and paw placement.

For footprint analysis, non-toxic red and blue inks were applied to the forepaws and hindpaws, respectively. Mice then walked along a narrow, paper-covered runway. Footprints were recorded and analyzed for stride length and width to evaluate gait patterns and motor coordination.

### 2.26 Statistical analysis

Data are presented as mean ± standard error of the mean (SEM) from at least three independent experiments. For comparisons between two groups, an unpaired two-tailed Student's *t*-test was applied. For comparisons among multiple groups at a single time point, one-way ANOVA followed by Tukey's post hoc test was used. For comparisons involving multiple groups across different time points (datasets with both group and time as factors), two-way ANOVA with Bonferroni's post hoc correction was performed. Statistical analyses and graph generation were conducted using GraphPad Prism 10.4 (San Diego, CA, USA). A p-value <0.05 was considered statistically significant.

## 3 Results

### 3.1 Macrophages phagocytosing MD after SCI undergo cellular senescence

**Fig. 1:**
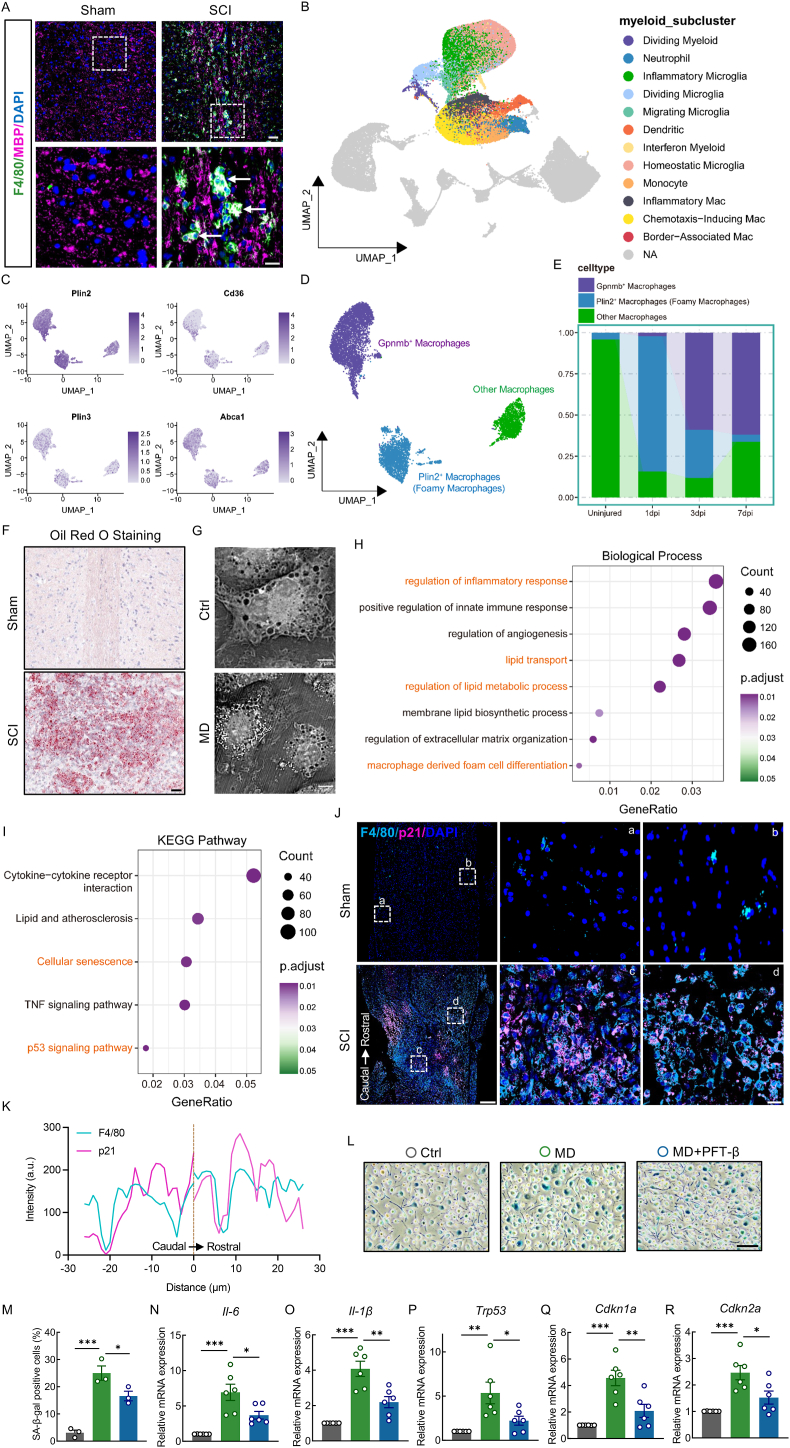
Macrophages phagocytosing MD after SCI undergo cellular senescence **A,** Representative IF labeling images of F4/80 (green) and MBP (pink) obtained from spinal cords in Sham and SCI mice at 7 dpi; scale bar = 50/25 μm. **B,** UMAP plot of all myeloid cells from uninjured spinal cord and 1, 3, and 7 dpi. Neutrophils, monocytes, macrophages, microglia, div-myeloid, and dendritic cells were extracted, reclustered, and re-embedded in new UMAP coordinates. Cells are colored by myeloid subtype as indicated in legend on right. **C,** Expression patterns of canonical marker genes, DEGs, and genes associated with disease are shown. Cells are colored based on their expression levels., with values represented as log-transformed normalized expression counts. **D,** Macrophages were extracted, reclustered, and re-embedded into new UMAP coordinates. Foamy macrophages were identified by *Plin2*, while *Gpnmb*^*+*^ macrophages were identified by *Gpnmb*. **E,** Proportion of each macrophages subtype among all macrophages at each time point. **F,** ORO staining showing lipid accumulation in spinal cords of Sham and SCI mice at 7 dpi; scale bar = 50 μm. **G,** Label-free panoramic live-cell imaging of BMDMs treated with MD (1 mg/mL) for 12 h; scale bar = 5 μm. **H,** GO biological process terms associated with the top DEGs between Control and MD group. GO biological process terms displayed along y axis, and generatio displayed along x axis. **I,** KEGG enrichment analysis of the DEGs for Control and MD group. Significant enrichment pathways between groups displayed along y axis, and generatio displayed along x axis. **J,** Representative IF labeling images of F4/80 (blue) and p21 (pink) in spinal cords from Sham and SCI mice at 7 dpi; scale bar = 200/20 μm. **K,** Co-localization analysis of F4/80 and p21 in macrophages of injured cords. **L,** SA-β-gal staining in BMDMs treated with MD (1 mg/mL) for 12 h following pre-treatment with PFT-β (10 μM, 24 h); scale bar = 100 μm. **M,** Quantification of β-gal-positive macrophages. **N–R,** Relative mRNA levels of SASP factors and senescence markers in BMDMs treated as above. ∗, p < 0.05, ∗∗, p < 0.01, and ∗∗∗, p < 0.001.

**Fig. S2:**
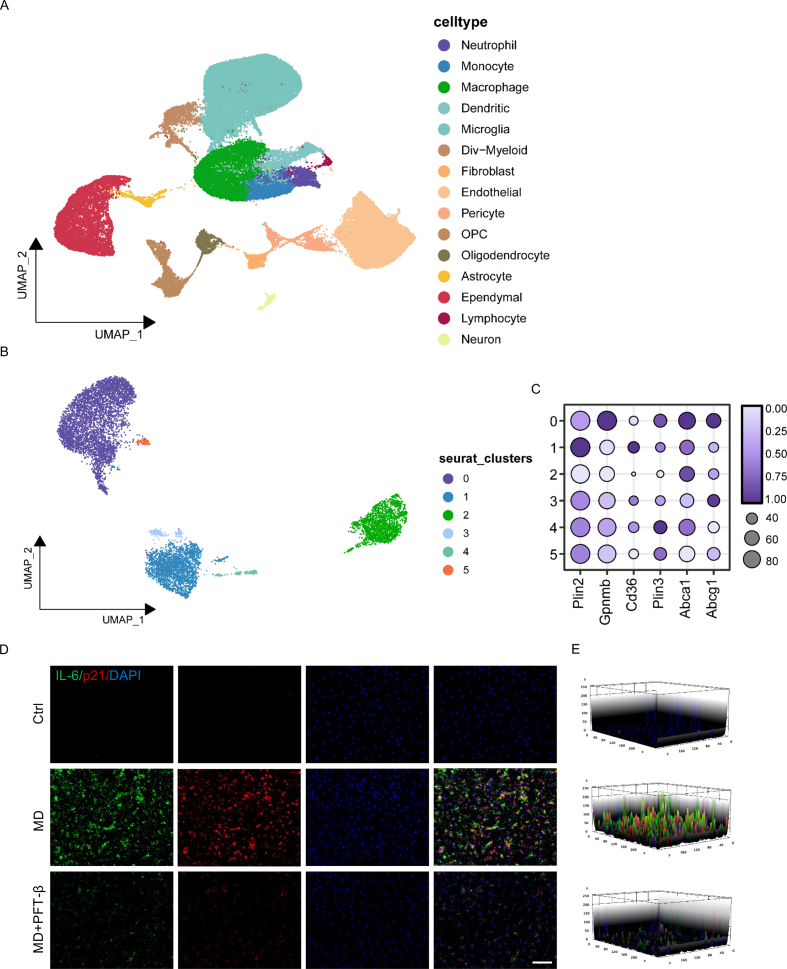
Foamy macrophages formed after macrophages engulf MD exhibit a senescent phenotype **A,** UMAP plot showing all cells from the uninjured spinal cord and injured spinal cord at 1, 3, and 7 dpi. Cells are colored and annotated by cell type based on a combination of DEGs, canonical markers, and previously published spinal cord scRNA-seq datasets. **B,** UMAP plot of macrophages from uninjured spinal cord and 1, 3, and 7 dpi. Macrophages were extracted, reclustered, and re-embedded in new UMAP coordinates. **C,** Dot plot displaying the expression pattern of the DEGs that best characterize each cell type. The intensity of dot color reflects the z-score of expression levels, and the size of the dot indicates the percentage of cells in which at least one UMI was detected for each gene. **D,** IF images showing IL-6 (green) and p21 (red) in BMDMs; scale bar = 100 μm. **E,** Quantitative of IL-6 and p21 fluorescence intensity.

To investigate the phagocytosis of MD by macrophages after SCI, IF staining was performed using F4/80 to label macrophages and myelin basic protein (MBP) to label MD. A strong co-localization of F4/80 and MBP was observed at the injury site in SCI mice (white arrows), but not in the Sham group. This suggested that macrophages extensively phagocytose MD in the injured region after SCI, performing the task of debris clearance (Fig. 1A). After phagocytosing MD, macrophages degrade the myelin components and release substantial amounts of lipids, such as cholesterol and phospholipids. Due to the limited metabolic capacity of macrophages, these lipids accumulate intracellularly, leading to the formation of lipid droplets—organelles that store neutral lipids within a hydrophobic core surrounded by a phospholipid monolayer [46]. We further performed scRNA-seq bioinformatics analysis to explore the formation and characterization of foamy macrophages after SCI. Following quality control, low-quality cells were removed, leaving a total of 66,178 cells for subsequent analysis. Cluster analysis of these remaining cells revealed 15 distinct clusters, each displaying a specific temporal progression when visualized using a UMAP plot. These 15 clusters encompassed all major cell types known to be present at the SCI site, including microglia, monocytes, macrophages, neutrophils, dendritic cells, astrocytes, oligodendrocytes, oligodendrocyte precursor cells, neurons, fibroblasts, and pericytes, among others (Fig. S2A). To better understand myeloid heterogeneity, we performed further cluster analysis of neutrophils, monocytes, macrophages, microglia, and dendritic cells and identified 12 distinct subtypes (Fig. 1B). To further identify foamy macrophages after SCI, macrophages were extracted and reclustered (Fig. S2B), as per the specific gene expression patterns (Plin2, Gpnmb, Cd36, Plin3, Abca1 and Abcg1) of these clusters (Fig. S2C and Fig. 1C), we identified three macrophage subtypes, which were labeled as Gpnmb^+^ macrophages, Plin2^+^ macrophages (characterized by foamy macrophages) and other macrophages (Fig. 1D–E). Therefore, the formation of foamy macrophages may represent a key process influencing the pathological outcomes after SCI. ORO staining revealed stronger lipid signals and abundant foam cell formation in the injured spinal cord of SCI mice compared to the Sham group (Fig. 1F). To further confirm these observations *in vitro*, BMDMs were isolated and co-cultured with purified MD. Live-cell imaging using an ODT microscope demonstrated the accumulation of numerous bright punctate structures—indicative of lipid droplets—in the cytoplasm of MD-treated BMDMs, compared to controls. These macrophages displayed a foam cell-like morphology (Fig. 1G). To investigate the biological processes affected by macrophages after engulfing MD, we performed Bulk RNA sequencing on foamy macrophages generated post-engulfment. The results showed that GO analysis was mainly enriched in biological processes such as inflammation regulation, lipid droplet transport and metabolism, and foam cell formation (Fig. 1H). Interestingly, KEGG analysis indicated that foamy macrophages after engulfing MD undergo cellular senescence and affect the p53 signaling pathway (Fig. 1I). To examine whether macrophages undergo senescence following MD phagocytosis, IF co-staining of F4/80 and the senescence marker p21 was performed. Increased co-localization of F4/80 and p21 was detected at the injury site of SCI mice, indicating that macrophages phagocytosing MD exhibit a senescent phenotype (Fig. 1J and K). To confirm the induction of senescence in foamy macrophages, BMDMs were treated with the p53-specific inhibitor PFT-β. SA-β-gal staining revealed a significant increase in β-gal-positive cells following MD treatment, which was attenuated by PFT-β, suggesting that p53 signaling is involved in MD-induced senescence (Fig. 1L and M). Moreover, qPCR analysis showed significant upregulation of senescence markers (p53, p21, and p16) and SASP factors (IL-6, IL-1β) in MD-treated macrophages, which were markedly reduced upon PFT-β treatment (Fig. 1N–R). IF also showed increased expression of IL-6 and p21 in MD-treated BMDMs, which was reversed by senescence inhibition (Fig. S2D and E). Collectively, these findings provide the first evidence that macrophages phagocytosing MD after SCI acquire a senescent phenotype.

### 3.2 MD-engulfing macrophages exhibit senescence-mediated efferocytosis dysfunction and aggravate astrocytic scarring and neuronal damage

**Fig. 2:**
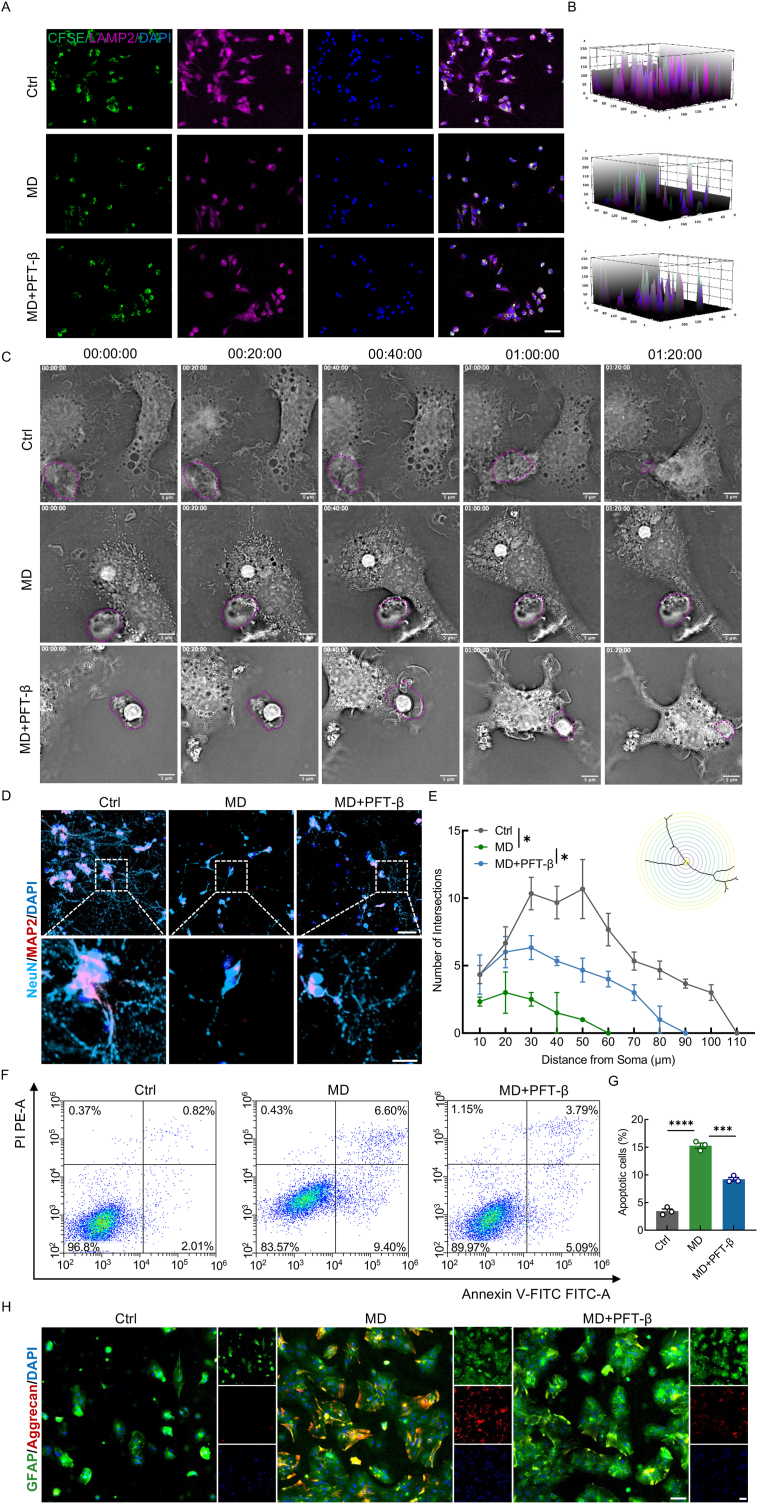
MD-engulfing macrophages exhibit senescence-mediated efferocytosis dysfunction and aggravate astrocytic scarring and neuronal damage **A,** Representative IF images of CFSE-labeled apoptotic neurons (green) and LAMP2 (pink) in BMDMs; scale bar = 50 μm. **B,** Quantification of CFSE and LAMP2 fluorescence intensity. **C,** Live-cell label-free imaging of BMDMs treated with MD and PFT-β; scale bar = 5 μm. **D,** IF images showing NeuN (blue) and MAP2 (red) in primary neurons co-cultured with BMDMs; scale bar = 50/25 μm. **E,** Quantitative Sholl analysis of neurite intersections. **F,** FCM plots of apoptotic neurons labeled with PI and annexin V-FITC. **G,** Quantification of apoptotic neuron percentages. **H,** IF images of GFAP (green) and Aggrecan (red) in primary astrocytes co-cultured with BMDMs.; scale bar = 100 μm ∗, p < 0.05, ∗∗, p < 0.01, and ∗∗∗, p < 0.001.

**Fig. S3:**
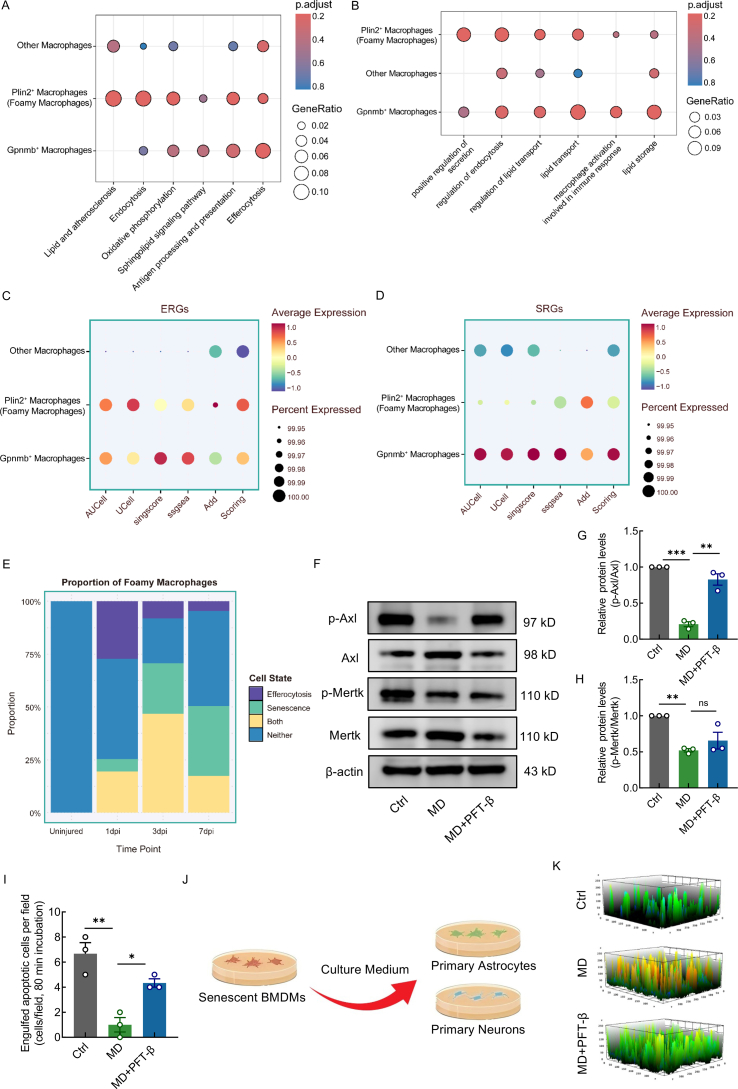
Macrophage senescence impairs efferocytosis and worsens glial and neuronal pathology **A-B,** KEGG and GO enrichment analysis of DEGs for each macrophage subtype. KEGG and GO biological process terms displayed along x axis, and the macrophage subtypes displayed along y axis. **C-D**, Bubble plots depict the activity score of ERGs and SRGs across macrophages subtypes, as determined by AUCell, UCell, singscore, ssGSEA, AddModulescore, and Scoring (the sum of scores from other algorithms). Color of dots represents z-scored expression level, and size of dots represents percentage of cells with at least one UMI detected per gene. **E**, The bar plot depicts the proportional composition of foamy macrophages in the efferocytosis, senescence or neither groups at each time point. The y-axis represents the proportion of each cell type, ranging from 0 to 1, while the x-axis distinguishes between the different time points. **F,** WB results for p-Axl and p-Mertk expression in BMDMs treated with MD (1 mg/mL) for 12 h after pre-treatment with PFT-β (10 μM) for 24 h; **G and H,** Densitometry analysis of p-Axl and p-Mertk expression. **I,** Quantitative analysis of the number of apoptotic neurons engulfed by BMDMs within 80 min in different groups. **J,** Schematic representation of the co-culture model involving BMDMs, primary neurons, and astrocytes. **K,** Quantitative analysis of fluorescence signal distribution for GFAP and Aggrecan in the co-culture system. ∗, p < 0.05, ∗∗, p < 0.01, and ∗∗∗, p < 0.001.

Through further analysis of the aforementioned three macrophage subtypes after SCI, functional enrichment revealed significant pathway differences across cell populations especially endocytosis and efferocytosis in foamy macrophage (Fig. S3A and B). Additionally, foamy macrophages exhibited high ERG and SRG activity (Fig. S3C and D). Moreover, as time progressed after SCI, the proportion of foamy macrophages exhibiting an efferocytic phenotype gradually decreased, while those exhibiting a senescent phenotype progressively increased (Fig. S3E). To assess whether efferocytosis is impaired in senescent macrophages after MD phagocytosis, we analyzed the expression and phosphorylation of the efferocytosis-related receptors Axl and Mertk. WB analysis showed a significant decrease in the phosphorylation levels of Axl and Mertk in MD-treated BMDMs compared to controls. Interestingly, PFT-β treatment restored Axl phosphorylation but had no significant effect on Mertk phosphorylation, suggesting that senescence specifically impairs Axl-mediated efferocytosis (Fig. S3F–H). To further confirm that senescence impairs efferocytosis, we examined the expression of lysosome-associated membrane protein 2 (LAMP2), a lysosomal membrane protein essential for phagosome–lysosome fusion and intracellular degradation [47]. CFSE-labeled apoptotic neurons (green fluorescence) were co-cultured with BMDMs from different treatment groups. IF revealed that although apoptotic cell uptake occurred in all groups, MD-treated BMDMs exhibited markedly reduced LAMP2 expression, indicating impaired phagolysosomal maturation and lysosomal dysfunction. Notably, LAMP2 levels were restored upon PFT-β treatment, suggesting that senescence contributes to efferocytosis failure through lysosomal impairment (Fig. 2A and B). Live-cell imaging over 80 min was used to directly visualize the efferocytosis process. In the control group, BMDMs rapidly recognized and engulfed apoptotic cells, completing the process within 20 min (Fig. 2C and Vid. S1). In contrast, MD-treated BMDMs exhibited a marked delay in initiating efferocytosis, failing to internalize apoptotic cells within the same period (Fig. 2C and Vid. S2). Importantly, this impairment was reversed by PFT-β pretreatment, restoring efferocytosis efficiency to near-control levels (Fig. 2C, Fig. S3I and Vid. S3). To evaluate the functional consequences of impaired efferocytosis, MCM from each BMDMs group were applied to primary neurons and astrocytes (Fig. S3J). IF staining and Sholl analysis revealed that neurons exposed to MD-MCM exhibited significantly reduced axonal length and branching, whereas these deficits were attenuated by PFT-β pretreatment (Fig. 2D and E). FCM further confirmed increased neuronal apoptosis in the MD-MCM group, which was significantly reduced in the PFT-β-MCM group (Fig. 2F and G). When the same MCMs were applied to primary astrocytes, IF staining showed elevated expression of Aggrecan—a marker of reactive astrocytes and glial scar formation—in the MD-MCM group. PFT-β pretreatment significantly reduced Aggrecan expression, suggesting that senescent macrophages exacerbate astrogliosis, which can be alleviated by senescence inhibition (Fig. 2H and Fig. S3K). Together, these findings indicate that macrophages exhibit senescence-mediated impairment of efferocytosis after engulfing MD. This dysfunction contributes to secondary injury by promoting neuronal apoptosis, inhibiting axonal regeneration, and enhancing astrocytic scar formation.

### 3.3 GLP-1R activation can inhibit macrophage senescence and efferocytosis impairment after MD engulfment

**Fig. 3:**
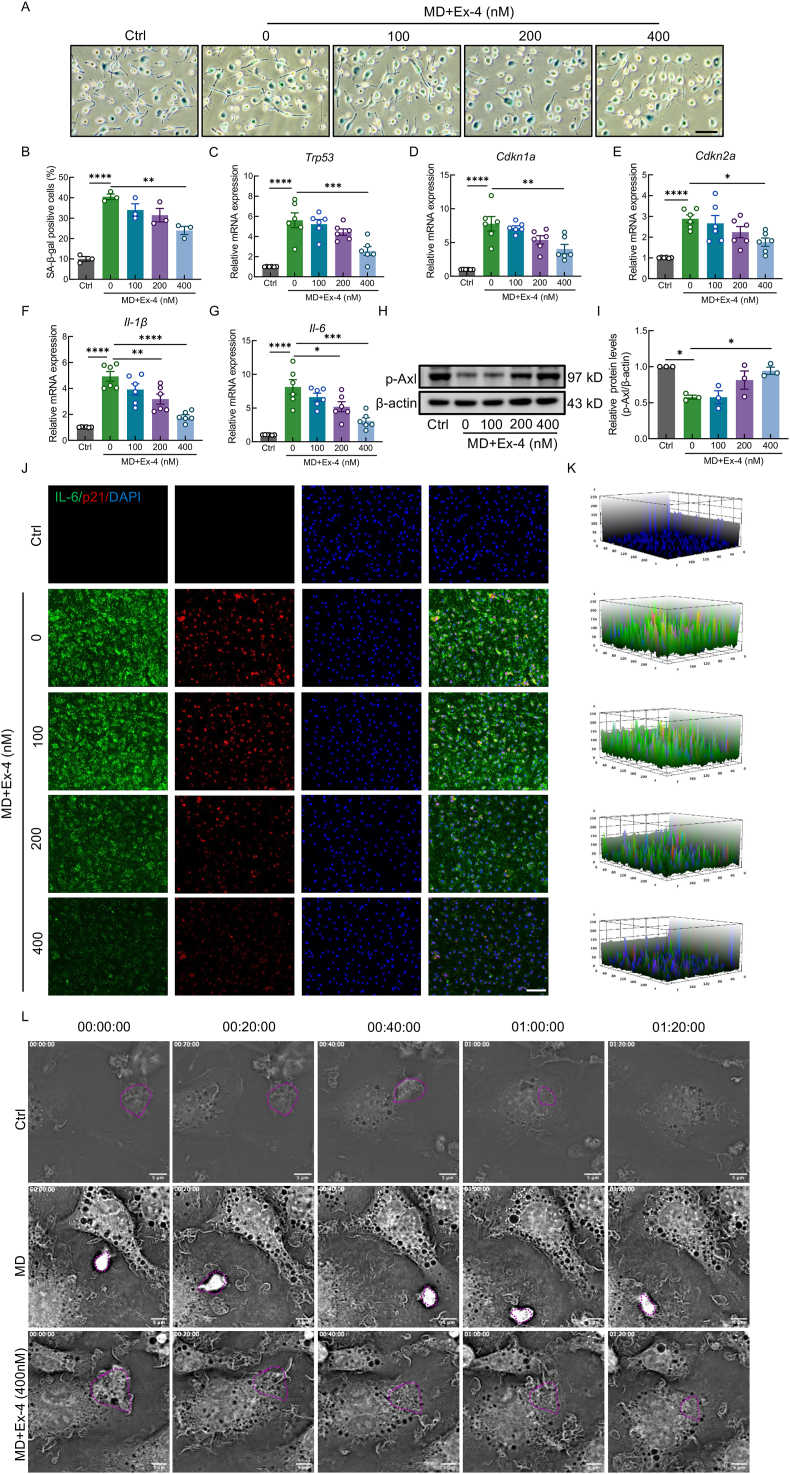
GLP-1R activation can inhibit macrophage senescence and efferocytosis impairment after MD engulfment **A,** SA-β-gal staining in BMDMs treated with MD and Ex-4; scale bar = 100 μm. **B,** Quantification of SA-β-gal-positive macrophages. **C–G,** Relative mRNA expression of SASP factors and senescence markers following Ex-4 treatment; **H,** WB analysis of p-Axl in BMDMs; **I,** Densitometric quantification of p-Axl. **J,** IF images showing IL-6 (green) and p21 (red) in BMDMs; scale bar = 100 μm. **K,** Quantification of IL-6 and p21 fluorescence. **L,** Label-free live-cell imaging of BMDMs; scale bar = 5 μm ∗, p < 0.05, ∗∗, p < 0.01, and ∗∗∗, p < 0.001.

**Fig. S4:**
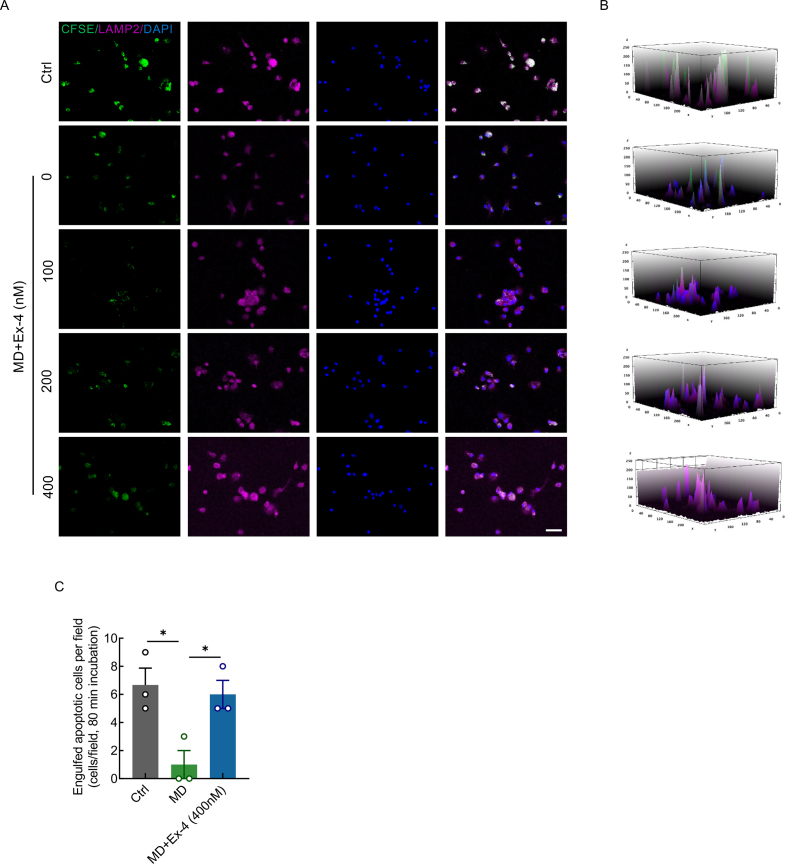
Ex-4 enhances efferocytosis in macrophages **A,** Representative IF images of CFSE-labeled apoptotic neurons (green) and LAMP2 (pink) in BMDMs after Ex-4 treatment; scale bar = 50 μm. **B,** Quantitative analysis of CFSE and LAMP2 fluorescence intensity. **C,** Quantification of apoptotic neurons engulfed by BMDMs within 80 min under different treatment conditions. ∗, p < 0.05, ∗∗, p < 0.01, and ∗∗∗, p < 0.001.

Recent studies have suggested that activation of the GLP-1R can modulate cellular senescence [48]. Our previous work demonstrated that the GLP-1R agonist Ex-4 significantly promoted neurological recovery following SCI in mice [35]. To investigate whether Ex-4 alleviates MD-induced macrophage senescence, BMDMs were treated with MD in the presence of Ex-4 at three different concentrations. SA-β-gal staining revealed a significant increase in senescent cells following MD treatment, while 400 nM Ex-4 markedly reduced the percentage of SA-β-gal-positive cells. No significant changes were observed with 100 or 200 nM Ex-4 compared to the MD group (Fig. 3A and B). QPCR analysis showed that 400 nM Ex-4 significantly downregulated the MD-induced mRNA expression of SASP factors IL-6 and IL-1β, as well as senescence regulators p53, p21, and p16 (Fig. 3C–G). IF staining further confirmed that Ex-4 treatment reduced IL-6 and p21 protein levels in BMDMs compared to the MD-only group (Fig. 3J and K). To determine whether Ex-4 restored efferocytosis, we assessed the phosphorylation of the efferocytosis-related receptors Axl. WB analysis revealed a significant reduction in p-Axl levels following MD exposure, which was notably restored by 400 nM Ex-4 treatment, suggesting that Ex-4 reactivates Axl-mediated efferocytosis in senescent macrophages (Fig. 3H and I). Additionally, IF analysis of BMDMs co-cultured with CFSE-labeled apoptotic neurons showed that Ex-4 restored the reduced LAMP2 expression observed in MD-treated macrophages, indicating improved lysosomal function and phagolysosomal fusion (Fig. S4A and B). Label-free live-cell imaging over an 80-min period demonstrated that BMDMs in the control group completed efferocytosis within 20 min after initial contact with apoptotic cells (Fig. 3L and Vid. S4). In contrast, MD-treated BMDMs failed to initiate engulfment during the same timeframe (Fig. 3L and Vid. S5). However, treatment with 400 nM Ex-4 enabled BMDMs to successfully complete the efferocytosis process (Fig. 3L, Fig. S4C and Vid. S6).

### 3.4 Gas6 is a critical downstream target of Ex-4 in alleviating macrophage senescence and efferocytosis impairment

**Fig. 4:**
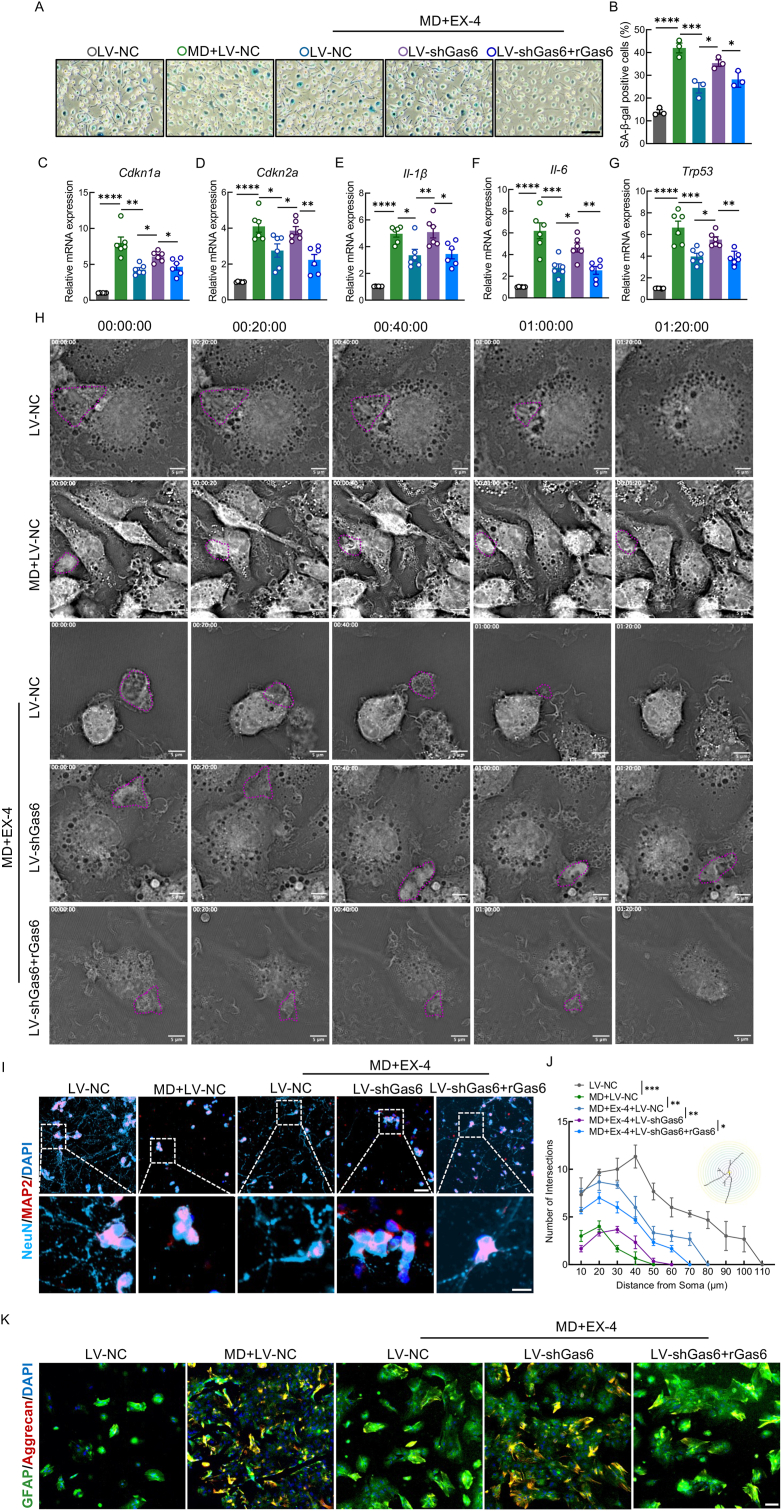
Gas6 is a critical downstream target of Ex-4 in alleviating macrophage senescence and efferocytosis impairment **A,** SA-β-gal staining in BMDMs transfected with LV-shGas6; scale bar = 100 μm. **B,** Quantification of SA-β-gal-positive cells. **C–G,** Relative mRNA expression of SASP factors and senescence markers in LV-shGas6-transfected BMDMs. **H,** Live-cell imaging of BMDMs post-LV-shGas6 transfection; scale bar = 5 μm. **I,** IF images of NeuN (blue) and MAP2 (red) in neurons co-cultured with Gas6-deficient BMDMs; scale bar = 50/25 μm. **J,** Sholl analysis of neuronal branching. **K,** IF images of GFAP (green) and Aggrecan (red) in astrocytes co-cultured with Gas6-deficient BMDMs; scale bar = 100 μm ∗, p < 0.05, ∗∗, p < 0.01, and ∗∗∗, p < 0.001.

**Fig. S5:**
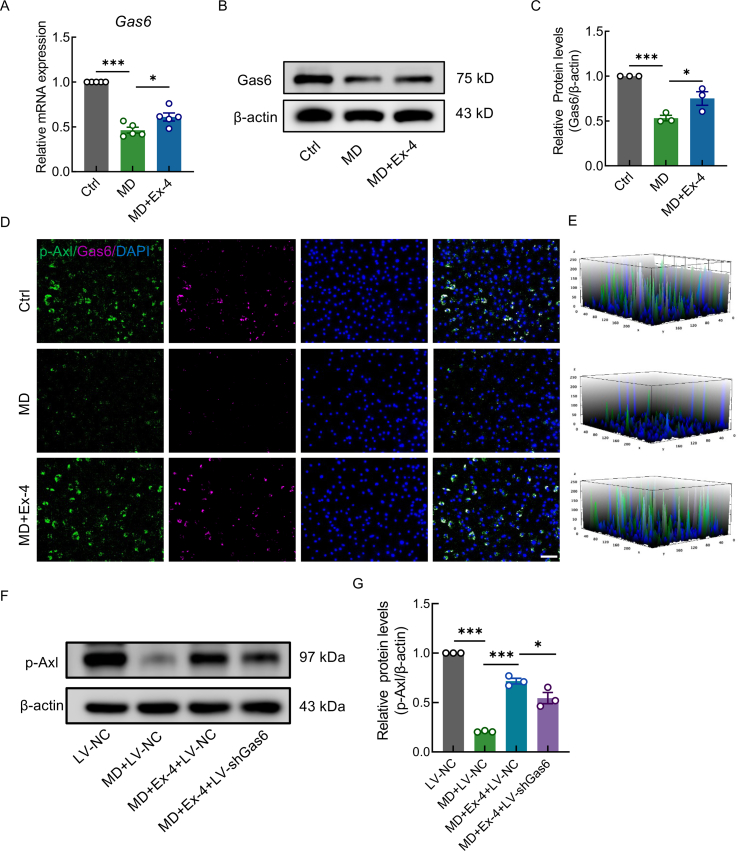
Ex-4 significantly activates Gas6 signaling in macrophages **A,** Relative mRNA expression of Gas6 in BMDMs after Ex-4 treatment. **B,** WB results for Gas6 expression in BMDMs after Ex-4 treatment. **C,** Densitometry analysis of Gas6 expression. **D,** Representative IF labeling images of p-Axl (green) and Gas6 (pink) in BMDMs after Ex-4 treatment; scale bar = 50 μm. **E,** Quantitative fluorescence intensity analysis of p-Axl and Gas6. **F,** WB results for p-Axl expression in BMDMs after transfection with LV-shGas6. **G,** Densitometric analysis of p-Axl protein levels. ∗, p < 0.05, ∗∗, p < 0.01, and ∗∗∗, p < 0.001.

**Fig. S6:**
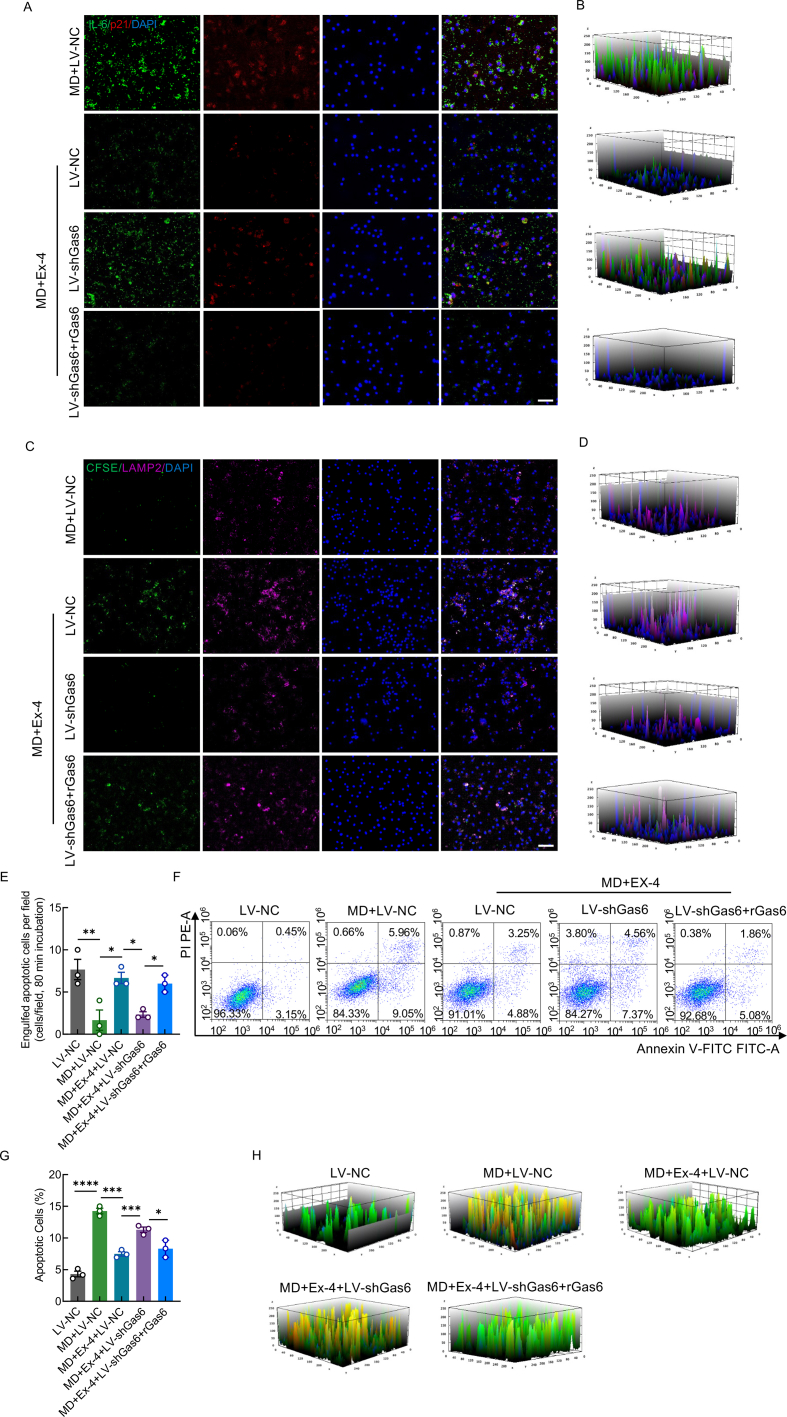
Gas6 knockdown impairs EX-4's effects on macrophage senescence and efferocytosis dysfunction **A,** Representative IF images of IL-6 (green) and p21 (red) in BMDMs after transfection with LV-shGas6; scale bar = 100 μm. **B,** Quantitative analysis of IL-6 and p21 fluorescence intensity. **C,** Representative IF labeling images of CFSE-labeled apoptotic neurons (green) and LAMP2 (pink) in BMDMs after transfection with LV-shGas6; scale bar = 50 μm. **D,** Quantitative fluorescence intensity of CFSE and LAMP2. **E,** Quantification of apoptotic neuron engulfment by BMDMs within 80 min under different conditions. **F,** FCM plots of neuronal apoptosis. **G,** Quantification of apoptotic neurons. **H,** Quantitative analysis of GFAP and Aggrecan fluorescence in the co-culture system. ∗, p < 0.05, ∗∗, p < 0.01, and ∗∗∗, p < 0.001.

To elucidate the molecular mechanisms by which GLP-1R activation mitigates macrophage senescence and efferocytotic dysfunction, 400 nM Ex-4 was selected for further investigation. Previous studies have identified Gas6, a vitamin K-dependent ligand of the receptor tyrosine kinase Axl, as a key mediator of anti-inflammatory responses and efferocytosis, contributing to tissue homeostasis [49,50]. Based on these findings, we assessed Gas6 expression in BMDMs. QPCR analysis revealed that MD treatment significantly downregulated Gas6 mRNA levels compared to controls, whereas Ex-4 treatment restored Gas6 transcription (Fig. S5A). WB analysis confirmed that the protein expression of Gas6 followed a similar trend (Fig. S5B and C). IF staining further demonstrated that Ex-4 reversed the MD-induced reduction in Gas6 and p-Axl expression, indicating that GLP-1R activation enhances the Gas6/Axl signaling pathway (Fig. S5D and E). To determine the functional relevance of Gas6 in Ex-4-mediated effects, we silenced Gas6 in BMDMs using LV-shGas6. Although Ex-4 increased p-Axl expression in MD-treated cells, this effect was abolished upon Gas6 knockdown (Fig. S5F and G). SA-β-gal staining demonstrated that Ex-4 treatment significantly decreased the proportion of senescent macrophages. While Gas6 suppression abolished this effect and led to an increase in senescent cells, the addition of rGas6 effectively rescued the phenotype, resulting in a renewed reduction of senescent macrophages. (Fig. 4A and B). Consistently, qPCR analysis revealed that Ex-4 significantly downregulated the MD-induced expression of SASP-related genes (IL-6, IL-1β), as well as senescence markers p53, p21, and p16. This inhibitory effect was abolished by Gas6 knockdown, which restored their elevated expression levels; however, supplementation with rGas6 effectively rescued the phenotype, again reducing the expression of these senescence-associated genes. (Fig. 4C–G). IF staining of IL-6 and p21 mirrored these findings, further supporting the role of Gas6 in mediating Ex-4's anti-senescent effects (Fig. S6A and B). To evaluate the impact of Gas6 on efferocytosis, we co-cultured BMDMs with apoptotic neurons. IF analysis showed that Ex-4 restored the MD-induced loss of LAMP2 expression, an effect that was abolished by Gas6 knockdown. Importantly, supplementation with rGas6 rescued this phenotype, leading to the recovery of LAMP2 expression levels. (Fig. S6C and D). Unlabeled live-cell imaging revealed that while BMDMs exposed to MD failed to initiate efferocytosis (Fig. 4H and Vid. S7, S8), Ex-4 treatment enabled successful engulfment of apoptotic cells within 40 min (Fig. 4H and Vid. S9). However, this pro-efferocytotic effect was abrogated in Gas6-silenced BMDMs, which remained incapable of completing efferocytosis within the same timeframe. Notably, supplementation with rGas6 restored efferocytotic capacity, enabling BMDMs to effectively complete efferocytosis. (Fig. 4H, Fig. S6E and Vid. S10, S11). We next investigated the neurological consequences of senescent macrophage after Gas6 silence. Sholl analysis and IF staining revealed that neurons cultured with MCM from MD-treated BMDMs exhibited significantly reduced axonal length and branching, accompanied by increased neuronal apoptosis as assessed by FCM (Fig. 4I and J, Fig.S6 F and G). Ex-4 pretreatment of BMDMs before MD exposure substantially preserved neuronal morphology and reduced apoptosis, indicating that Ex-4 attenuates macrophage senescence-induced neurotoxicity. However, this neuroprotective effect was lost following Gas6 knockdown. Importantly, supplementation with rGas6 restored the neuroprotective effect, as evidenced by preserved neuronal integrity and reduced apoptosis, highlighting the essential role of Gas6-mediated efferocytosis in this process (Fig. 4I and J, Fig.S6 F and G). Furthermore, IF analysis of astrocytes cultured with MCM revealed elevated expression of Aggrecan when exposed to MCM from MD-treated BMDMs. Ex-4 pretreatment significantly reduced Aggrecan expression, whereas Gas6 knockdown reversed this effect. Notably, supplementation with rGas6 restored the suppressive effect on Aggrecan expression, indicating that Gas6-dependent efferocytosis can mitigate astrocytic scar formation. (Fig. 4K and Fig. S6H). Collectively, these findings demonstrate that MD-induced macrophage senescence not only impairs efferocytosis but also contributes to neuroglial dysfunction—including neuronal damage and astrocytic scarring—and that these pathological effects can be reversed by Ex-4 through a Gas6-dependent mechanism.

### 3.5 Ex-4 attenuates macrophage senescence and efferocytosis dysfunction through activating Gas6 in SCI mice

**Fig. 5:**
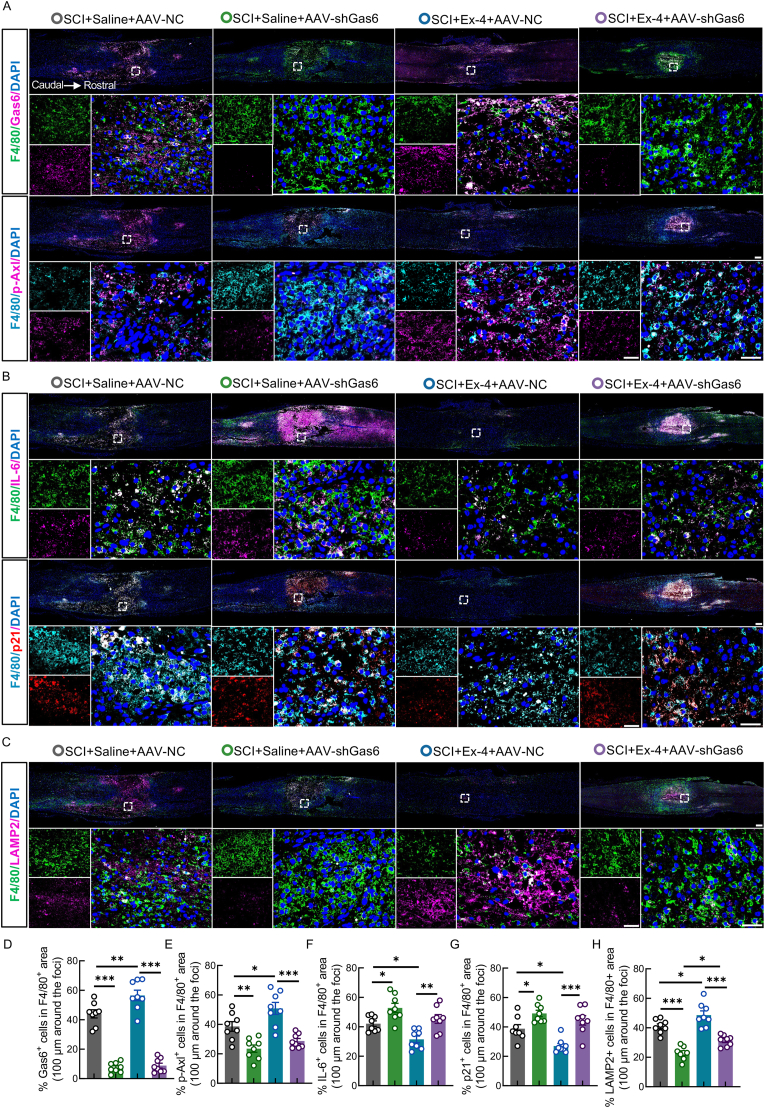
Ex-4 attenuates macrophage senescence and efferocytosis dysfunction through activating Gas6 in SCI mice **A,** IF images of F4/80 (green), Gas6 (pink), F4/80 (blue), p-Axl (pink) at 7 dpi in spinal cords following AAV-shGas6 and Ex-4 treatment; scale bar = 200/100/50 μm. **B,** IF images of F4/80 (green), IL-6 (pink), F4/80 (blue), p21 (red); scale bar = 200/100/50 μm. **C,** IF images of F4/80 (green) and LAMP2 (pink); scale bar = 200/100/50 μm. **D–H,** Quantification of Gas6-, p-Axl-, IL-6-, p21-, and LAMP2-positive macrophage areas. ∗, p < 0.05, ∗∗, p < 0.01, and ∗∗∗, p < 0.001.

To validate the *in vitro* findings *in vivo*, we utilized AAV-shGas6 to knock down Gas6 expression in a mouse SCI model and evaluated its role in Ex-4–mediated regulation of macrophage senescence and efferocytosis. IF analysis showed that Ex-4 markedly increased Gas6 expression in macrophages at the injury site, whereas AAV-shGas6 administration significantly reduced Gas6 levels, confirming efficient AAV-mediated Gas6 knockdown (Fig. 5A and D). Notably, Gas6 inhibition significantly decreased p-Axl expression compared to the injury-only group. Although Ex-4 treatment upregulated p-Axl expression relative to the injury group, this effect was abolished when Ex-4 was combined with Gas6 knockdown (Fig. 5A and E). These findings confirm that Ex-4 enhances Axl activation via Gas6, underscoring Gas6 as a key upstream effector of the Axl-mediated efferocytotic response. We next evaluated macrophage senescence and efferocytosis markers in the injured spinal cord. IF staining revealed increased expression of SASP markers IL-6 and p21 in macrophages from the AAV-shGas6 group compared to the injury-only group (Fig. 5B–F and G). Ex-4 treatment significantly reduced IL-6 and p21 levels, consistent with its anti-senescence effects; however, this benefit was negated in the presence of Gas6 knockdown (Fig. 5B–F and G). Furthermore, the Gas6 knockdown group exhibited markedly reduced LAMP2 expression at the lesion site, indicating impaired efferocytotic capacity (Fig. 5C and H). While Ex-4 treatment enhanced LAMP2 expression compared to the injury-only group, this enhancement was significantly attenuated when Gas6 was silenced, suggesting that Ex-4–induced efferocytosis depends critically on Gas6 (Fig. 5C and H). In summary, consistent with *in vitro* findings, Ex-4 suppresses macrophage senescence and restores efferocytosis after SCI via activation of the Gas6/Axl pathway. These protective effects are contingent upon the presence of Gas6, highlighting its essential role in mediating the therapeutic action of Ex-4.

### 3.6 Ex-4 suppresses glial scarring and promotes remyelination and axonal regeneration in SCI mice via the Gas6/Axl signaling pathway

**Fig. 6:**
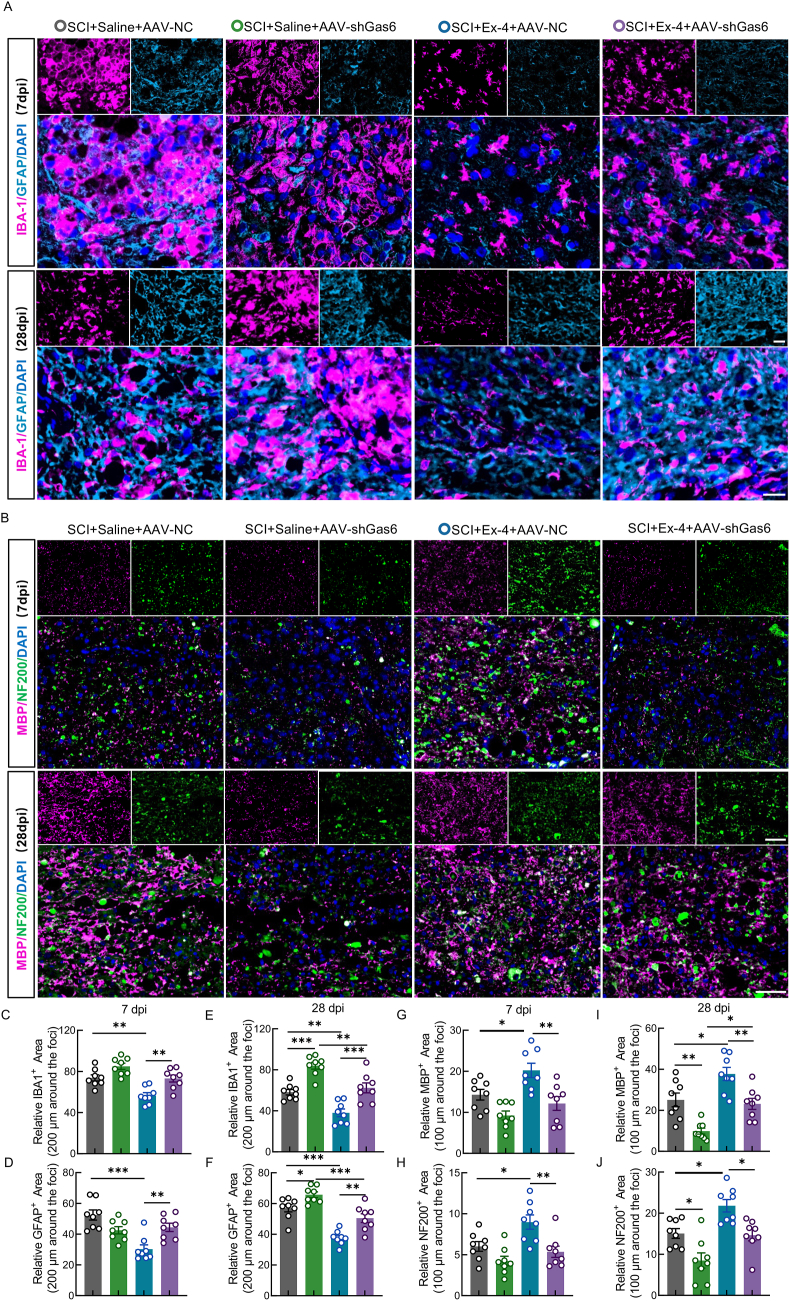
Ex-4 suppresses glial scarring and promotes remyelination and axonal regeneration in SCI mice via the Gas6/Axl signaling pathway **A,** IF images of IBA-1 (pink) and GFAP (blue) at 7 and 28 dpi; scale bar = 100/50 μm. **B,** IF images of MBP (pink) and NF-200 (green); scale bar = 100/50 μm. **C and D,** Quantification of glial scar area at 7 dpi. **E and F**, Glial scar area at 28 dpi. **G and H,** Quantification of myelinated and axonal area at 7 dpi. **I and J,** Myelinated and axonal area at 28 dpi. ∗, p < 0.05, ∗∗, p < 0.01, and ∗∗∗, p < 0.001.

Activation of IBA-1^+^ microglia and GFAP^+^ astrocytes is a hallmark of the inflammatory response following SCI. These glial cells proliferate and migrate toward the lesion core, contributing to glial scar formation—a dense extracellular matrix that releases inhibitory molecules and impedes axonal regeneration [51]. Based on this, we further investigated the effects of Ex-4 and Gas6 modulation on glial scar formation in SCI mice. IF analysis revealed no significant difference in the IBA1^+^ and GFAP^+^ areas between the SCI-only and Gas6 knockdown groups at 7 days post-injury (dpi) (Fig. 6A–C and D). However, by 28 dpi, Gas6 inhibition resulted in a marked increase in both IBA1^+^ and GFAP^+^ areas compared to the SCI-only group (Fig. 6A–E and F). In contrast, Ex-4 treatment significantly reduced the extent of IBA1^+^ and GFAP^+^ staining at both 7 and 28 dpi, indicating a suppression of glial scar formation (Fig. 6A and C–F). Notably, this inhibitory effect of Ex-4 was reversed upon Gas6 knockdown, suggesting that the anti-scarring effects of Ex-4 depend on Gas6 signaling (Fig. 6A and C–F). Since remyelination stabilizes regenerating axons and axonal regrowth provides a substrate for myelin repair, we next assessed both processes simultaneously. MBP and neurofilament 200 (NF-200) were used to evaluate remyelination and axonal regeneration, respectively. IF results showed no significant difference in MBP^+^ and NF-200^+^ areas between the SCI-only and Gas6 knockdown groups at 7 dpi, but significant reductions were observed in the Gas6 knockdown group by 28 dpi (Fig. 6B and G–J). In contrast, Ex-4 treatment significantly increased both MBP^+^ and NF-200^+^ areas, particularly at 28 dpi, indicating enhanced remyelination and axonal regeneration (Fig. 6B–I and J). However, these beneficial effects of Ex-4 were largely abolished when Gas6 expression was silenced (Fig. 6B–I and J). These results demonstrate that Ex-4 mitigates glial scarring and facilitates remyelination and axonal regrowth after SCI, and these therapeutic effects are critically dependent on the presence of Gas6.

### 3.7 Ex-4 promotes neuroprotection and functional recovery in SCI mice through the Gas6/Axl axis

**Fig. 7:**
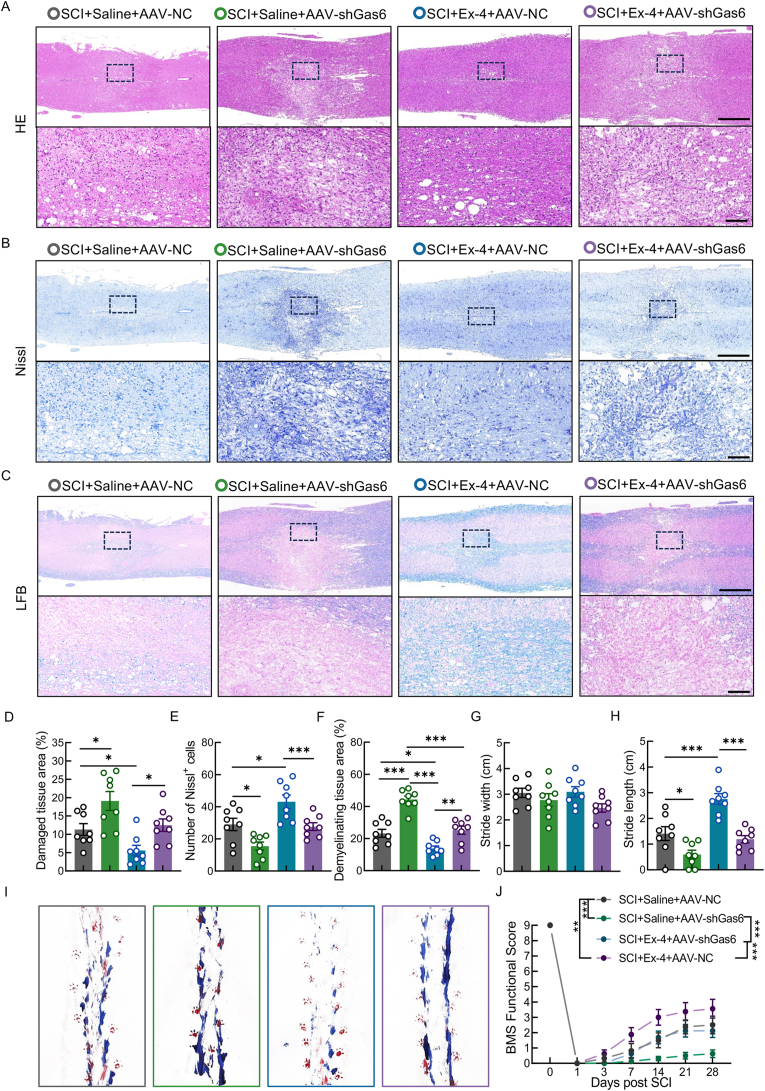
Ex-4 promotes neuroprotection and functional recovery in SCI mice through the Gas6/Axl axis **A,** HE staining of spinal cords at 28 dpi; scale bar = 500/100 μm. **B,** Nissl staining at 28 dpi; scale bar = 500/100 μm. **C,** LFB staining at 28 dpi; scale bar = 500/100 μm. **D-F,** Quantification of lesion size, neuronal survival, and demyelination. **G and H,** Footprint analysis quantification at 28 dpi. **I,** Representative footprint images at 28 dpi. **J,** BMS scores across 28 dpi. ∗, p < 0.05, ∗∗, p < 0.01, and ∗∗∗, p < 0.001.

To further evaluate the therapeutic efficacy of Ex-4 in SCI, neurohistological analyses and behavioral assessments were performed, focusing on the involvement of the Gas6/Axl signaling axis. HE staining revealed that the Gas6 knockdown group exhibited a significantly larger lesion area compared to the injury-only group, with disrupted gray and white matter structures, partial dissolution of nerve fibers, tissue loosening, edema, and vacuolar degeneration (Fig. 7A and D). In contrast, Ex-4 treatment markedly reduced the lesion area, indicating enhanced neural tissue preservation. However, this neuroprotective effect was attenuated in the Ex-4 + AAV-shGas6 group, suggesting the necessity of Gas6 in mediating Ex-4's protective effects (Fig. 7A and D). Nissl staining showed a notable reduction in the number and intensity of Nissl bodies in the Gas6 inhibition group, indicative of neuronal damage (Fig. 7B and E). The number of Nissl bodies was significantly increased in the Ex-4 treatment group compared to the injury-only group, whereas this effect was reversed upon Gas6 knockdown (Fig. 7B and E). LFB staining, used to assess myelin integrity, showed that Gas6 inhibition significantly reduced the extent of remyelination in the lesion area (Fig. 7C and F). In contrast, Ex-4 treatment significantly enhanced remyelination, but this effect was largely diminished in the presence of AAV-shGas6 (Fig. 7C and F), further supporting the role of the Gas6/Axl pathway in Ex-4-mediated myelin repair. Functional recovery was assessed using hindlimb footprint analysis and the BMS scores. Although no significant differences in step width were observed, the Gas6 inhibition group showed reduced stride length, paw dragging, and irregular gait patterns compared to the injury-only group (Fig. 7G–I). Ex-4-treated mice demonstrated longer strides and more coordinated gait, while these improvements were reversed in the Ex-4 + AAV-shGas6 group (Fig. 7G–I). Consistently, BMS scores were significantly higher in Ex-4-treated mice than in the injury-only group, reflecting improved motor function (Fig. 7J). However, BMS scores declined upon Gas6 inhibition, indicating impaired functional recovery (Fig. 7J). Taken together, these findings demonstrate that Ex-4 effectively attenuates neuropathological damage and promotes motor function recovery in SCI mice, and these therapeutic effects are critically dependent on the activation of the Gas6/Axl signaling pathway.

### 3.8 AMPK phosphorylation is required for Ex-4-induced activation of the Gas6/Axl pathway

**Fig. 8:**
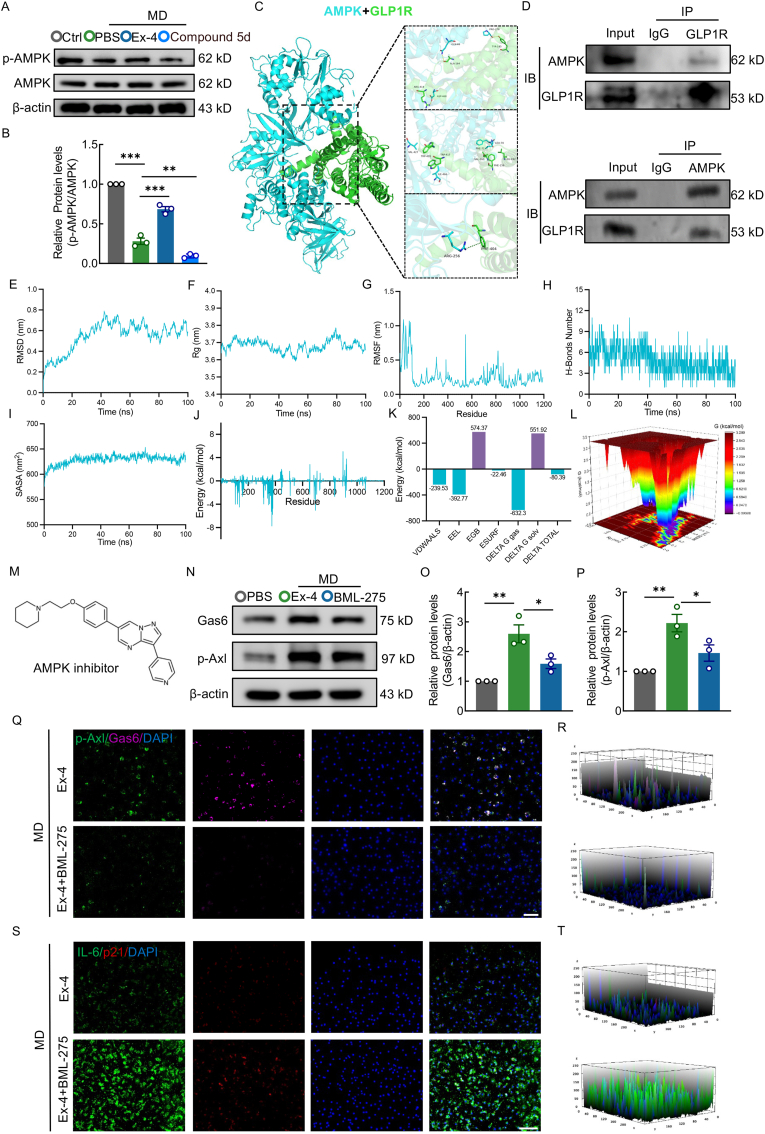
AMPK phosphorylation is required for Ex-4-induced activation of the Gas6/Axl pathway **A,** WB for p-AMPK and AMPK in BMDMs treated with MD, Ex-4 and Compound 5D. **B,** Densitometry analysis of p-AMPK. **C,** Molecular docking model of GLP-1R and AMPK. **D**, Co- IP for GLP-1R and AMPK. **E-G**, RMSD, Rg, and RMSF analyses of the GLP-1R–AMPK complex. **H and I,** Hydrogen bond counts and SASA of the complex. **J and K,** MM/GBSA energy contribution of key residues. **L,** FEL analysis of the complex. **M,** Chemical structure of BML-275. **N,** WB of Gas6 and p-Axl after BML-275 treatment. **O and P,** Densitometric quantification of Gas6 and p-Axl. **Q,** IF images of p-Axl (green) and Gas6 (pink) in BMDMs; scale bar = 100 μm. **R**, Quantification of fluorescence intensity. **S,** IF of IL-6 (green) and p21 (red) in BMDMs; scale bar = 100 μm. **T,** Quantification of IL-6 and p21. ∗, p < 0.05, ∗∗, p < 0.01, and ∗∗∗, p < 0.001.

Our previous data established that Ex-4 exerts therapeutic effects in SCI through the GLP-1R/Gas6/Axl signaling axis. However, the precise intracellular mechanisms linking GLP-1R activation to Gas6 upregulation remain elusive. Prior studies have demonstrated that GLP-1R can elevate intracellular cyclic AMP levels upon ligand binding, subsequently activating protein kinase A (PKA), which in turn may phosphorylate and activate AMPK [52,53]. To investigate this potential intermediate step, we first evaluated AMPK activation following Ex-4 treatment. WB analysis revealed a significant increase in the p-AMPK/AMPK ratio in macrophages, indicating Ex-4-induced phosphorylation of AMPK. Interestingly, treatment with the GLP-1R inhibitor Compound 5D alone reduced basal AMPK phosphorylation (Fig. 8A and B). To further probe the molecular interactions between GLP-1R and AMPK, in silico protein–protein docking was performed. The docking score was −405.30, indicating a strong binding affinity (Fig. 8C). Hydrogen bonds were observed between GLP-1R residues Tyr145(A), Ala164(A), and Arg414(A) and AMPK residues Pro278(A), Gln66(A), and Asp468(A). Hydrophobic interactions involved GLP-1R residues Phe156(A), Val160(A), Leu192(A), Trp417(A), and Trp420(A) with AMPK residues Leu70(A), Phe71(A), Ile466(A), and Val427(A). A notable cation–π interaction was formed between Phe404(A) of GLP-1R and Arg256(A) of AMPK (Fig. 8C). We next performed Co-IP in BMDMs. The results revealed an interaction between GLP-1R and AMPK (Fig. 8D), suggesting that GLP-1R may associate with AMPK to mediate downstream signaling, which supports the notion that GLP-1R activation contributes to AMPK phosphorylation and the subsequent modulation of macrophage efferocytosis and senescence. To validate the stability of this protein complex, a 100 ns molecular dynamics simulation was conducted. The RMSD fluctuated during the initial 0–75 ns but subsequently stabilized (Fig. 8E). The Rg remained around 3.7 nm, suggesting a compact and stable protein conformation (Fig. 8F). RMSF analysis indicated that residues within the binding interface fluctuated within 0.4 nm, reflecting local stability (Fig. 8G). Hydrogen bond analysis showed 0–11 stable hydrogen bonds throughout the simulation, and the SASA remained steady at ∼625 nm^2^ (Fig. 8H and I). MM/GBSA analysis yielded a binding free energy of −80.39 kcal/mol, with major contributing residues including ARG375, GLU379, GLN371, ARG695, TRP381, ARG136, PHE351, GLN106, PHE122, and LEU315 (Fig. 8J and K). The free energy landscape (FEL) further confirmed stable conformations within the RMSD range of 0–0.65 nm and Rg range of 3.65–3.78 nm (Fig. 8L). These results collectively support a stable and strong interaction between GLP-1R and AMPK, suggesting that GLP-1R activation may directly induce AMPK phosphorylation. To validate whether AMPK activation is required for the downstream activation of the Gas6/Axl signaling pathway, we treated macrophages with the AMPK inhibitor BML-275 (Fig. 8M). WB analysis showed that although Ex-4 significantly increased Gas6 and Axl expression levels, the addition of BML-275 markedly attenuated this effect (Fig. 8N–P), suggesting AMPK is indispensable for Ex-4-mediated Gas6/Axl pathway activation. IF results in BMDMs further corroborated these findings (Fig. 8Q and R). Finally, we investigated whether AMPK inhibition would reverse Ex-4's suppression of macrophage senescence. IF analysis demonstrated that the addition of BML-275 significantly increased IL-6 and p21 expression levels compared to Ex-4 treatment alone (Fig. 8S and T), indicating that Ex-4′s anti-senescent effects were AMPK-dependent. Together, these data indicate that Ex-4 modulates macrophage senescence and efferocytosis dysfunction following SCI via the GLP-1R/AMPK/Gas6/Axl signaling axis.

## 4 Discussion

Macrophage senescence is a pathological state characterized by irreversible cell cycle arrest, altered metabolic activity, and secretion of pro-inflammatory and tissue-degrading factors, collectively known as SASP [30]. Macrophage senescence is driven by complex molecular mechanisms involving various signaling pathways, transcriptional regulators, and metabolic changes [54]. Persistent DNA damage activates the DNA damage response, stabilizing p53 and upregulating p21, while the p16INK4a-Rb pathway reinforces cell cycle arrest [55,56]. Studies have shown that bone marrow-derived macrophage senescence can drive dysfunction in distant tissues, leading to secondary pathological conditions [57]. However, the occurrence and underlying mechanisms of macrophage senescence following SCI remain unexplored and unreported.

In the present study, IF analysis revealed that macrophages in the injured area of mice following SCI expressed high levels of SASP components such as IL-6, simultaneously with p21 showing strong co-localization with macrophages. This finding suggested that macrophages rapidly entered a senescent state as an early response to SCI. Traditional theories attribute macrophage senescence to age-related cellular changes, and many studies have utilized aged mice to isolate and culture senescent macrophages [[58], [59], [60]]. However, the observed macrophage senescence in the early stages of SCI in 6–8-week-old mice was evidently independent of age-associated degenerative changes. Therefore, the study focus was shifted to the pathological processes associated with secondary changes following SCI. Mechanical trauma after SCI leads to extensive axonal damage, oligodendrocyte death, and demyelination, resulting in the release of MD at the injury site [7,61]. MD contains inhibitory molecules, such as neurite outgrowth inhibitor A, myelin-associated glycoprotein, and oligodendrocyte myelin glycoprotein, which suppress axonal regeneration and cause growth cone collapse and cytoskeletal destabilization [62,63]. Additionally, MD acts as a pro-inflammatory stimulus, triggering macrophages and microglia via receptors like Toll-like receptor 4 to release cytokines, such as TNF-α, IL-6, and IL-1β, and creating a chronic inflammatory environment that exacerbates secondary damage [64]. Macrophage phagocytosis of MD is a critical step in promoting tissue repair following SCI. However, excessive MD uptake leads to their transformation into foam cells [65]. Key receptors, such as scavenger receptor class A and CD36, recognize and internalize MD, leading to the accumulation of lipids and cholesterol within the macrophages, which results in the formation of lipid droplets, a defining characteristic of foam cells [66]. In the present study, ORO staining experiments and unlabeled live cell imaging of macrophages after MD phagocytosis confirmed this phenomenon. Thus, we sought to determine whether foam cell formation induced by MD phagocytosis was causally linked to macrophage senescence. SA-β-gal staining, IF, WB, and qPCR results compared to those of the control group outcomes confirmed that macrophages that phagocytosed MD showed a significant increase in senescence-specific markers, including β-galactosidase and SASP expression. Interestingly, the expression of these markers decreased when a specific p53 inhibitor was used to block the senescence process. This indicated that the senescence phenotype formed by macrophages after MD phagocytosis was similar to the traditional senescence phenotype, while also possessing its own specific characteristics. This phenotype is thus a novel finding in the context of SCI.

Senescent macrophages are characterized by cellular dysfunction, including impaired ability to perform effective phagocytosis [67]. One key aspect of this dysfunction is the failure of senescent macrophages to clear apoptotic cells and cellular debris, a process known as efferocytosis [68], which is critical for tissue repair and inflammation resolution post-SCI. At the molecular level, senescent macrophages exhibit a decline in the expression of key efferocytosis receptors, such as Axl, which are involved in recognizing and engulfing apoptotic cells. In addition, the increase in SASP factors, including IL-6 and IL-1β, further exacerbates the efferocytosis dysfunction by inducing chronic inflammation and obstructing proper phagocytic activity. Moreover, senescent macrophages may also show impaired actin cytoskeleton dynamics, leading to a reduced ability to effectively engulf apoptotic cells [69]. These alterations impair inflammation resolution and promote chronic inflammation, which is associated with various pathological conditions, including tissue fibrosis and neurodegeneration [70]. The present research revealed that macrophage ability to perform efferocytosis is significantly impaired after they undergo senescence following MD phagocytosis. This is primarily evidenced by a notable reduction in the expression of the efferocytosis receptor Axl as well as a significant decrease in the expression of LAMP2, which is responsible for the macrophages' phagocytic and digestive functions. Furthermore, label-free live-cell panoramic imaging technology revealed that senescent macrophages lose the ability to engulf surrounding dead cells within the same time span. Interestingly, their efferocytotic function is restored when senescence is inhibited. Here, we report novel evidence that senescent macrophages after SCI exhibit efferocytosis dysfunction. As a result, identifying and exploring therapeutic drugs and key targets that promote efferocytosis in senescent macrophages has become a major focus of the current research.

GLP-1R is a G protein-coupled receptor primarily found in the pancreas, brain, heart, and gastrointestinal tract [34,71]. It plays a significant role in glucose homeostasis regulation, insulin secretion, and various metabolic processes [72]. It also plays a crucial role in macrophage senescence regulation via several molecular mechanisms, which have been explored in atherosclerosis [73]. In our previous work, as an agonist of GLP-1R, Ex-4 alleviated microglia-mediated neuroinflammation following SCI by modulating the PI3K/ARAP3/RhoA signaling pathway [35]. However, whether Ex-4 regulates macrophage senescence and promotes efferocytosis after SCI has not been established. Subsequent experiments demonstrated that treatment with 400 nM Ex-4 markedly reduced the expression of senescence markers, such as β-gal and SASP, thereby blocking macrophage senescence after MD phagocytosis, and also effectively promoted Axl-mediated efferocytosis.

After SCI, the inflammatory microenvironment significantly influences the activation state of glial cells and the survival of neurons. In the co-culture system of senescent macrophages with astrocytes and neurons, we observed that senescent macrophages strongly promoted astrocyte gliosis and neuronal apoptosis. However, when Ex-4-treated macrophages were introduced into the system, there was a marked suppression of pro-inflammatory astrocytic gliosis, as well as enhanced neuronal survival and neurite outgrowth.

To further investigate the specific molecular mechanisms and downstream targets by which Ex-4 regulated macrophage senescence and promoted efferocytosis after SCI, previous studies indicated that Gas6 may play a crucial role in this process. Gas6 is a vitamin K-dependent protein that is a key player in several physiological processes, including cell survival, proliferation, migration, and immune modulation [74,75]. It is involved in various cellular signaling pathways, particularly through its interaction with receptors, such as Axl, MerTK and Tyro3. Among these, Gas6 primarily signals through the Axl receptor [76,77]. Gas6 binds to the extracellular domain of Axl and triggers its dimerization and autophosphorylation on tyrosine residues [78]. This activates several signaling pathways, including the PI3K/Akt pathway, which promotes cell survival and metabolism [79]. Gas6/Axl signaling in macrophages enhances efferocytosis by increasing phagocytic receptor expression and promoting an anti-inflammatory M2 phenotype [80]. Additionally, Gas6 has been shown to counteract the senescence process by preventing SASP factor expression [81]. Therefore, LV and AAV were used to further regulate Gas6 expression to clarify the molecular mechanism underlying the effects of Ex-4. Both *in vitro* and *in vivo* results showed that although Ex-4 significantly inhibited macrophage senescence and promoted Axl-mediated efferocytosis, p-Axl expression was significantly reduced when Gas6 transcription was suppressed, and the regulatory effects of Ex-4 on these macrophage functions also disappeared. This indicated that the anti-senescence and efferocytosis-promoting effects of Ex-4 after SCI depend on the presence of Gas6 as a key downstream target.

Importantly, we further demonstrated that this neuroprotective effect of Ex-4 is critically dependent on the expression of Gas6. In our co-culture experiments, when Gas6 expression was silenced in macrophages, the ability of Ex-4 to suppress astrocyte activation and protect neurons was significantly diminished. This indicates that the anti-inflammatory and neuroprotective effects of Ex-4 are not solely due to its direct impact on macrophage senescence or efferocytosis, but also require Gas6 as a key mediator. Mechanistically, Gas6 likely contributes to modulating the cytokine secretion profile of macrophages, thereby indirectly shaping the astrocyte phenotype and neuronal viability.

Thus, the presence of Gas6 is essential for Ex-4 to exert its full regulatory function within the glia-neuron-macrophage triad. This highlights Gas6 not only as a downstream effector of Ex-4 in macrophages, but also as a critical molecular link influencing the broader neuroimmune microenvironment following SCI.

The possible pathways through which GLP-1R activation regulates Gas6 expression were further considered. Previous studies reported that AMPK was activated by GLP-1R through a multifaceted signaling cascade [82]. GLP1R stimulates the Gαs subunit upon activation by its ligand, leading to increased cAMP production and subsequent PKA activation [83]. PKA phosphorylates upstream kinases, such as LKB1, which directly activates AMPK by phosphorylation at Thr172 [84]. Alternatively, GLP-1R can increase intracellular calcium levels through a PLC/IP3 pathway, thereby activating another AMPK upstream kinase CaMKKβ [85]. The present *in vitro* studies with the Ex-4 treatment confirmed that GLP-1R activation significantly enhances AMPK phosphorylation. AMPK inhibitor was utilized to investigate whether AMPK influences Gas6 expression. The activation effects of Ex-4 on Gas6 and p-Axl were abolished under these conditions. Concurrently, the protective effects of Ex-4 against macrophage senescence and efferocytosis impairment were markedly diminished. These findings validated the critical role of the GLP-1R/AMPK/Gas6/Axl pathway in regulating macrophage senescence and efferocytosis following SCI. Therefore, this signaling axis represents a pivotal mechanism underlying the therapeutic effects of Ex-4 in this context.

While the glial scar triggered by microglia and astrocyte proliferation isolates the lesion and limits secondary damage after SCI, its inhibitory molecules prevent axonal sprouting and synaptic reconnection, impeding neural tissue remyelination [86]. Therefore, the formation of glial scars, neuropathological changes, and motor function recovery in SCI mice were further assessed to visually observe the therapeutic effects of Ex-4 and the key target Gas6 on SCI. The research results indicated that the Ex-4 treatment significantly reduced the glial scar area and decreased the size of the local injury area. It also promoted neuronal survival and axonal regeneration and induced remyelination. Furthermore, Ex-4-treated mice showed more regular gait, increased stride length, and reduced foot dragging. Their BMS scores were also higher, suggesting better motor function recovery. However, motor function recovery in mice was blocked regardless of the Ex-4 treatment when Gas6 was inhibited. This indicated that Gas6 expression itself had a profound impact on the pathological process of SCI and suggested that the therapeutic effect of Ex-4 was, to some extent, dependent on the presence of Gas6.

Although our study was performed in murine SCI models and primary macrophages, the findings have potential relevance to human disease. Clinical and pathological observations in SCI patients have consistently reported persistent myelin debris accumulation and chronic neuroinflammation, both of which represent major obstacles to neural repair [87]. These phenomena are highly consistent with the pathological context modeled in our experiments, supporting the translational value of our mechanistic findings. From a therapeutic perspective, GLP-1R agonists are already approved for the treatment of type 2 diabetes and obesity, with well-established safety and pharmacokinetic profiles in humans [88]. This clinical availability substantially lowers the barrier for repurposing Ex-4 in SCI, compared with novel agents that require de novo safety evaluation. Our results therefore not only identify macrophage senescence as a potential pathological contributor to SCI but also highlight Ex-4 as a promising therapeutic candidate for future translational studies. To bridge the gap between preclinical discovery and clinical application, further validation using human macrophages and exploratory clinical trials will be essential. However, there were several limitations in the present study. First, the concept that macrophages phagocytosing MD after SCI exhibit a senescence phenotype, along with senescence-induced efferocytosis impairment, is a novel idea proposed in the study. Further experiments are needed to validate it and explore its underlying mechanisms in greater depth. Second, the precise molecular pathways downstream of Gas6, particularly in relation to neuronal survival and repair, remain unclear, warranting further mechanistic exploration. In addition to the GLP-1R/AMPK/Gas6/Axl axis, other signaling pathways may also contribute to macrophage senescence and functional impairment following SCI. For example, pathways involved in autophagy, mTOR signaling, and inflammatory regulation have been reported to influence macrophage polarization, efferocytosis, and foam cell formation [89]. While our study primarily focused on the GLP-1R/AMPK/Gas6/Axl pathway, future work exploring these additional mechanisms could provide a more comprehensive understanding of macrophage regulation in SCI. Integrating such pathways may also reveal synergistic therapeutic targets to further enhance neural repair and functional recovery. Additionally, Ex-4 was administered intraperitoneally (20 μg/kg), resulting in systemic exposure, so the observed neuroprotective effects likely reflect both direct and systemic actions. Similar intraperitoneal administration has been used in SCI models to improve neuronal survival and functional recovery [90]. Future studies with intrathecal delivery or conditional knockouts could clarify local versus systemic contributions, while the current approach reflects clinically relevant systemic treatment. Moreover, it should be noted that direct validation using human spinal cord tissue is currently challenging due to the limited availability of surgical or postmortem specimens and the considerable ethical constraints associated with human sample collection. Despite these limitations, persistent myelin debris accumulation, chronic neuroinflammation, and impaired repair processes have been well-documented in patients with SCI, consistent with the pathological features modeled in our murine system. Beyond that, the GLP-1R/AMPK/Gas6/Axl signaling axis is evolutionarily conserved, although subtle species-specific differences in receptor expression or downstream responses may exist. Future studies employing human macrophages derived from SCI patients, as well as early-phase clinical investigations, will be crucial to further validate and translate these findings to human SCI.

In conclusion, our study provides new insights into how macrophages phagocytosing MD after SCI undergo senescence and exhibit impaired efferocytosis, thereby contributing to astrocytic gliosis and hindering neural recovery. As a GLP-1R agonist, Ex-4 activates the GLP-1R/AMPK/Gas6/Axl signaling pathway to suppress macrophage senescence, enhance efferocytosis, and thereby promote neural tissue regeneration, ultimately facilitating functional recovery in SCI mice. Future efforts should focus on translating these findings into human models through clinical trials and humanized models, aiming to establish GLP-1R agonists as viable therapeutic agents for SCI recovery.

## CRediT authorship contribution statement

**Mingjie Xia:** Funding acquisition, Investigation, Methodology, Writing – original draft. **Chaochen Li:** Data curation. **Yanan Zhang:** Investigation. **Tianyi Wang:** Investigation. **Chaoqiang Zhang:** Software. **Jian Zhou:** Methodology. **Xuexian Zhu:** Visualization. **Hongxiang Hong:** Validation. **Haijun Li:** Funding acquisition. **Zhanyang Qian:** Conceptualization, Funding acquisition. **Zhiming Cui:** Funding acquisition, Supervision, Writing – review & editing.

## Ethical approval

All experiments involving animals were conducted according to the ethical policies and procedures approved by the Ethics Committee of Nantong First People's Hospital in accordance with the Basel Declaration (Approval No. S20230727-007, Date: July 27, 2023). The Ethics Committee of Nantong First People's Hospital acts on the International Council for Laboratory Animal Science (ICLAS).

## Declaration of competing interest

The authors declare that they have no known competing financial interests or personal relationships that could have appeared to influence the work reported in this paper.

## Data Availability

The data from this study are available from the author for correspondence upon reasonable request.

## Abbreviations

AAV: Adeno-associated virus; AMPK: adenosine monophosphate-activated protein kinase; BMDMs: bone marrow-derived macrophages; BMS: Basso Mouse Scale; cDNA: complementary DNA; DMEM: Dulbecco's Modified Eagle Medium; Ex-4: exendin-4; Gas6: growth arrest-specific 6; GLP-1R; glucagon-like peptide-1 receptor; HE: Hematoxylin and Eosin; IF: Immunofluorescence; LAMP2: lysosome-associated membrane protein 2; LFB: Luxol Fast Blue; LV: Lentivirus; MBP: myelin basic protein; MD: myelin debris; ODT: optical diffraction tomography; ORO: Oil Red O; qPCR: real-time quantitative PCR; SASP: senescence-associated secretory phenotype; SCI: Spinal cord injury; SEM: standard error of the mean; sh: short hairpin; TNF-α: tumor necrosis factor-alpha; IL-1β: interleukin-1 beta.
