# Supplementary material for: GLP-1R activation restores Gas6-driven efferocytosis in senescent foamy macrophages to promote neural repair
Source: Redox Biol. 2025 Sep 3;86:103857. doi: 10.1016/j.redox.2025.103857 (PMC12446625; doi:10.1016/j.redox.2025.103857)
Supplement: Multimedia component 1 [file mmc1.docx]

**Supplementary Materials and Methods**

***1. Animals***

All animal experiments were conducted in strict accordance with ethical guidelines and regulations approved by the Ethics Committee of Nantong First People’s Hospital (Approval No. S20230727-007, Date: 27 July 2023), following the principles outlined in the Basel Declaration and in compliance with the standards of the International Council for Laboratory Animal Science (ICLAS). Every effort was made to minimize animal suffering and to reduce the number of animals used while maintaining scientific rigor.

Adult male C57BL/6 mice (20–25 g, 8–10 weeks old) were obtained from Huachuang Sino Pharma Tech Co., Ltd., Taizhou, China (License No. SCXK 2020-0009). Mice were acclimated for 7 days prior to experimentation in a specific pathogen-free (SPF) animal facility with controlled environmental conditions: temperature 22 ± 3°C, relative humidity 40–70%, and a 12-hour light/dark cycle (lights on at 07:00).

Animals were housed in standard polycarbonate cages with sterilized bedding, rodent chow, and autoclaved drinking water provided ad libitum. Cage cleaning and disinfection were performed twice weekly, and animals were monitored daily for signs of distress, abnormal behavior, or illness. Health status was recorded, and any mice showing signs of disease or stress were excluded from experiments in accordance with humane endpoints.

During all procedures, mice were handled gently to minimize stress, and all surgical interventions were performed under deep anesthesia using isoflurane or appropriate injectable anesthetics, with post-operative monitoring and analgesia provided according to institutional protocols.

The overall experimental design, including randomization, group allocation, and timing of interventions, is illustrated schematically in Fig. S1. Sample sizes were determined based on prior studies and power calculations to ensure adequate statistical reliability, and all animal procedures were performed blinded to experimental group assignments to minimize bias.

***2. Mouse Spinal Cord Injury (SCI) Model Establishment***

A standardized mouse spinal cord contusion injury model was established following previously published protocols, with modifications to ensure reproducibility and animal welfare. Adult male C57BL/6 mice (20–25 g, 8–10 weeks old) were used. Prior to surgery, mice were fasted for 4–6 hours but had free access to water. Surgical anesthesia was induced via intraperitoneal injection of a ketamine (100 mg/kg) and xylazine (20 mg/kg) mixture, achieving deep anesthesia confirmed by absence of the pedal withdrawal reflex and corneal reflex. Body temperature was maintained at 37°C using a heating pad throughout the procedure.

The surgical area, encompassing the thoracic vertebrae (T8–T12), was shaved and disinfected sequentially with 70% ethanol and povidone-iodine. A midline skin incision (~1.5–2 cm) was made along the dorsal midline to expose the underlying paraspinal muscles. Muscles were carefully separated along the spinous processes using fine blunt forceps, minimizing bleeding and avoiding excessive tissue trauma, until the T10 vertebra was fully exposed.

A laminectomy was performed at the T10 vertebral level using a microsurgical bone rongeur under a stereomicroscope to expose the dorsal surface of the spinal cord while preserving the dura mater. Care was taken to avoid direct mechanical injury during bone removal.

A controlled contusion injury was induced using a pneumatic-electronic precision impactor (68099Ⅱ, RWD, Shenzhen, China). The impactor parameters were set to deliver a consistent force and displacement, ensuring reproducible injury severity across animals. Successful SCI was confirmed by the appearance of a central spinal cord hematoma, a tail-flick reflex during impact, and complete hindlimb paralysis immediately post-injury.

Following injury, the incision was closed in layers: paraspinal muscles were sutured with absorbable 5-0 sutures, and the skin was closed using 4-0 nylon sutures. Mice were placed on a warming pad and monitored until full recovery from anesthesia. Postoperative care included administration of analgesics (e.g., buprenorphine 0.05 mg/kg, subcutaneously, every 12 h for 48 h) and antibiotics if necessary to prevent infection.

To assist bladder function, manual bladder expression was performed twice daily for 7–10 days or until spontaneous urination returned. Animals were housed individually for the first 24–48 h post-surgery to prevent injury by cage mates and were monitored daily for signs of distress, autophagy, or surgical site infection. Detailed surgical procedures, injury parameters, and postoperative care protocols were standardized and documented to ensure reproducibility.

***3. Single-cell RNA sequencing (scRNA-seq) Bioinformatics Data Acquisition***

To investigate cellular heterogeneity and gene expression dynamics following spinal cord injury (SCI), publicly available single-cell RNA sequencing (scRNA-seq) datasets were obtained from the Gene Expression Omnibus (GEO) database. Specifically, data from wild-type C57BL/6 mice were retrieved, encompassing normal (uninjured) spinal cord samples and injured samples at 1, 3, and 7 days post-injury (dpi). The GEO accession number for this dataset is GSE162610. Raw count matrices, metadata files, and cell-type annotations were downloaded and organized according to sample, time point, and experimental condition.

Prior to downstream analysis, gene expression matrices were filtered to remove low-quality cells. Cells expressing fewer than 200 genes or with >10% mitochondrial gene content were excluded. Gene expression normalization, scaling, and dimension reduction were performed using the Seurat (v4.3.0) R package, following standard scRNA-seq preprocessing pipelines.

To focus on biological processes relevant to our study, senescence-related genes (SRGs) were compiled by querying the Mouse Genome Informatics (MGI) database using the keyword “senescence”. This search retrieved genes with experimentally validated or predicted roles in cellular senescence, including markers of replicative, stress-induced, and organismal senescence.

Similarly, efferocytosis-related genes (ERGs) were identified through a combined query of the GeneCards database and MGI, using the keyword “efferocytosis”. Genes were included if they were annotated as regulators, mediators, or effectors of apoptotic cell clearance, phagocytosis, or macrophage-specific engulfment processes. Cross-referencing between databases ensured comprehensive coverage and minimized false negatives.

The resulting SRG and ERG gene lists were curated, checked for redundancy, and are provided in Table S1. These gene lists served as input for subsequent analyses, including differential gene expression, gene set enrichment, and cell-type-specific pathway analysis. All bioinformatics analyses were performed under a standardized workflow to ensure reproducibility and transparency.

***4. Single-Cell Subtype Classification and Annotation in SCI***

Following quality control and preprocessing of the scRNA-seq datasets, single-cell subtype classification and annotation were conducted using the Seurat (v4.3.0) R package following established best practices.

1. Data Normalization and Scaling:
   Raw gene expression matrices were normalized using the LogNormalize method in Seurat, where gene counts for each cell were divided by the total counts, multiplied by a scaling factor of 10,000, and log-transformed. Highly variable genes (HVGs) were identified using the FindVariableFeatures function with the “vst” method, selecting the top 2,000 genes for downstream analyses.
2. Data Transformation and Integration:
   To reduce batch effects across multiple samples and time points (0, 1, 3, 7 dpi), data were integrated using Seurat’s reciprocal PCA (RPCA) integration workflow. This procedure aligns cells across conditions while preserving biological variance. Post-integration, the data were scaled using ScaleData, regressing out cell cycle effects, mitochondrial gene percentage, and nUMI counts to minimize technical confounders.
3. Dimensional Reduction and Clustering:
   Principal component analysis (PCA) was performed on the scaled expression matrix, and the top 30 principal components (PCs) were used for downstream analyses. Cell neighborhoods were identified using FindNeighbors with a k-nearest neighbor (kNN) parameter of 20, and clustering was conducted via the Louvain algorithm (FindClusters) with a resolution parameter set between 0.4 and 1.2 to optimize granularity of subtypes.
4. Cluster Marker Identification:
   Marker genes for each cluster were identified using the FindAllMarkers function. This function compares gene expression in a given cluster against all other clusters using the Wilcoxon rank-sum test. Genes with log fold-change > 0.25 and adjusted P-value < 0.05 were considered significant. For pairwise comparisons between subclusters of the same cell type, FindMarkers was applied with similar criteria to identify subtype-specific differentially expressed genes (DEGs).
5. Cluster Annotation:
   Each cluster was annotated based on known canonical markers from literature, previously published SCI single-cell datasets, and the identified DEGs. For example, macrophage subtypes were distinguished using markers such as F4/80, Cd11b, Cd68, and microglia subtypes by Cx3cr1 and Tmem119. Neuronal and glial subtypes were annotated using markers including Map2, NeuN, Gfap, and Olig2. Annotation was cross-validated with CellMarker and other single-cell databases to ensure accuracy.
6. Visualization:
   Uniform Manifold Approximation and Projection (UMAP) was used for dimensional reduction and visualization of cellular distributions. The relative proportions of each cell type and subtype were calculated and visualized as bar plots, pie charts, or stacked UMAP plots to depict temporal changes post-injury.
7. Functional Enrichment Analysis:
   To explore the functional roles of each subtype, DEGs were subjected to Gene Ontology (GO) Biological Process and Kyoto Encyclopedia of Genes and Genomes (KEGG) pathway enrichment analyses using the clusterProfiler R package. An adjusted P-value < 0.05 (Benjamini-Hochberg correction) was used as the threshold for statistical significance. Enrichment results were visualized using dot plots, bar plots, and network diagrams to highlight key biological processes and signaling pathways associated with each cell subtype.
8. Reproducibility and Documentation:
   All scripts, parameters, and processed datasets were documented to ensure full reproducibility of the subtype classification and functional annotation pipeline. Random seeds were set (set.seed(1234)) for stochastic processes to ensure consistency across analyses.

***5. Scoring of SRG and ERG Activity Across Different Cell Types***

To quantitatively evaluate the activity of senescence-related genes (SRGs) and efferocytosis-related genes (ERGs) across diverse spinal cord cell populations, we implemented a multi-method scoring framework leveraging five complementary approaches: AUCell, singscore, UCell, single-sample Gene Set Enrichment Analysis (ssGSEA), and Seurat’s AddModuleScore. Each method captures different aspects of gene set activity at the single-cell level, providing a robust and reproducible assessment.

1. AUCell (v1.18.0):
   AUCell computes the Area Under the Curve (AUC) for the ranked expression of gene sets in each cell. Briefly, genes in each cell were ranked by expression levels, and the cumulative distribution of SRGs or ERGs was used to calculate the AUC. Cells in the top percentile of AUC values were considered highly active for the gene set. The threshold for active cells was determined automatically using the ‘AUCell_exploreThresholds’ function.
2. singscore (v1.12.0):
   The singscore method provides a rank-based, single-sample scoring approach. For each cell, genes were ranked according to normalized expression values, and scores were computed based on the relative ranks of SRGs or ERGs. The score for each cell represents its relative enrichment in the gene set, scaled between –1 and 1, with higher scores indicating stronger activity.
3. UCell (v1.3.0):
   UCell applies a rank-based scoring algorithm optimized for single-cell data. For each cell, it calculates a normalized enrichment score by comparing the ranks of gene set members against the background distribution of all expressed genes. UCell is particularly robust to sparse expression and dropout events in scRNA-seq datasets.
4. ssGSEA (implemented via GSVA v1.46.0):
   Single-sample Gene Set Enrichment Analysis (ssGSEA) computes an enrichment score per cell by integrating the empirical cumulative distribution of gene expression ranks for the target gene set versus all other genes. This approach captures relative up- or downregulation of SRGs or ERGs in individual cells. Parameters used included method="ssgsea" and kcdf="Gaussian" for continuous expression distributions.
5. Seurat AddModuleScore (v4.3.0):
   Using Seurat’s AddModuleScore function, we calculated average expression scores of SRGs or ERGs for each cell, after subtracting aggregated control feature sets randomly sampled from genes with similar expression levels. This normalization accounts for differences in library size and global expression trends.

Visualization and Interpretation:

1. The resulting scores from all five methods were projected onto UMAP embeddings generated in Seurat to visualize spatial distribution of SRG or ERG activity across clusters.
2. Cells with high SRG scores were color-coded in warmer tones (e.g., red), indicating heightened senescence-related activity, while those with high ERG scores were similarly highlighted.
3. Comparative analyses across cell types were performed to identify which populations exhibited peak SRG or ERG activity at different post-injury time points.
4. Concordance among the five scoring methods was evaluated to ensure robustness, with Spearman correlation coefficients calculated across methods for each cell.

Reproducibility:

1. Random seeds were set (set.seed(1234)) for stochastic components in AUCell and AddModuleScore calculations.
2. All parameters, thresholds, and code for scoring and visualization were documented to enable full reproducibility.

This multi-method scoring framework allowed us to reliably quantify cell type-specific senescence and efferocytosis activity, providing a high-resolution map of functional gene set dynamics during spinal cord injury progression.

***6. Adeno‑Associated Virus (AAV) Vector Packaging and Injection***

To achieve macrophage-specific knockdown of Gas6, a short hairpin RNA (shRNA) sequence targeting the Gas6 transcript (5′-GCTCAGTGACTATGCTTAA-3′) was cloned into an adeno-associated virus (AAV) vector under the control of the F4/80 promoter, which ensures selective expression in macrophages. A scrambled shRNA sequence with no known homology to the mouse genome was cloned into the same vector and used as a negative control (AAV-NC). All constructs were verified by Sanger sequencing prior to viral packaging.

The recombinant vectors were packaged into AAV serotype 9 (AAV9) by GeneChem, Shanghai, China, with a final viral titer of 1.33 × 10¹² viral genomes (vg)/mL, quantified using quantitative PCR. Aliquots were stored at –80°C to maintain viral stability and avoid repeated freeze-thaw cycles.

Experimental Design and Randomization:

Adult male C57BL/6 mice (20–25 g) were randomly assigned to three experimental groups: (1) Sham, (2) AAV-NC, and (3) AAV-shGas6, with at least n = 6 mice per group. Investigators performing injections, behavioral assessments, and subsequent molecular analyses were blinded to the viral group allocation to minimize bias.

Systemic AAV Delivery:

Prior to injection, mice were gently restrained in a custom mouse restrainer to minimize stress and movement. The lateral tail vein was chosen for systemic delivery due to its accessibility and high transduction efficiency. Tails were first disinfected using 70% ethanol and allowed to air-dry for 1–2 minutes.

Using a 30-gauge insulin syringe, a total volume of 200 μL of viral solution (1.33 × 10¹² vg/mL) was slowly injected into the lateral tail vein over 60–90 seconds to prevent reflux and local vein damage. Following injection, gentle pressure was applied at the injection site with sterile gauze for 30–60 seconds to ensure hemostasis.

Post-Injection Monitoring:

Mice were monitored continuously for 1 hour post-injection for immediate adverse reactions, including signs of distress, tail necrosis, or abnormal locomotion. Thereafter, mice were observed daily for at least 7 days to record body weight, general activity, grooming, and any signs of systemic toxicity or inflammation at the injection site.

Verification of Transduction Efficiency:

In preliminary experiments, macrophage-specific expression of the viral construct was validated by qPCR, Western blot, and immunofluorescence staining for Gas6 in isolated bone marrow-derived macrophages (BMDMs) and spinal cord tissue. Only viral preparations demonstrating >70% knockdown efficiency in macrophages were used for subsequent in vivo experiments.

Safety and Ethical Considerations:

All viral procedures were performed under biosafety level 2 (BSL-2) conditions, with appropriate personal protective equipment (PPE). All experimental protocols were approved by the Ethics Committee of Nantong First People’s Hospital (Approval No. S20230727-007), and mice were housed in specific pathogen-free conditions with controlled temperature (20–26°C), humidity (40–70%), and a 12-h light/dark cycle.

***7. In Vivo Exendin-4 (Ex-4) Treatment***

To investigate the therapeutic effects of Exendin-4 (Ex-4) on spinal cord injury (SCI) recovery, adult male C57BL/6 mice (20–25 g) subjected to SCI were randomly assigned to either the Ex-4 treatment group or a vehicle control group, with at least n = 6 mice per group. All behavioral and histological assessments were performed by investigators blinded to the treatment groups to minimize bias.

Preparation of Ex-4 Solution:

Lyophilized Ex-4 powder (HY-13443, MedChemExpress, USA) was reconstituted in sterile phosphate-buffered saline (PBS, pH 7.4) to obtain a stock solution of 50 µg/mL. Aliquots were stored at –20°C and protected from light. Before each injection, an appropriate volume of stock was diluted to working concentration so that the administered dose was 20 µg/kg body weight, calculated based on the most recent body weight of each mouse. Fresh working solutions were prepared daily to maintain stability and biological activity.

Intraperitoneal Injection Procedure:

Mice were gently restrained using a soft restrainer to minimize stress. The abdominal area was inspected and, if necessary, wiped with 70% ethanol to reduce contamination. Ex-4 was administered via intraperitoneal (i.p.) injection using a sterile insulin syringe with a 30-gauge needle. The total injection volume did not exceed 100 µL per mouse to prevent abdominal discomfort or leakage.

Injections were performed once daily at the same time (±30 min) each day for seven consecutive days post-SCI to ensure consistency and reduce circadian-related variability in drug metabolism and pharmacodynamics. The timing was coordinated with behavioral assessment schedules to avoid acute drug effects confounding functional readouts.

Post-Injection Monitoring:

Immediately following injection, mice were observed for 5–10 minutes for signs of distress, abdominal swelling, lethargy, or abnormal posture. Thereafter, mice were monitored daily for weight changes, food and water intake, general activity, and grooming behavior. Any adverse events were recorded, and humane endpoints were predefined according to institutional animal care guidelines.

Controls and Experimental Design:

The vehicle control group received an equal volume of sterile PBS via intraperitoneal injection at the same schedule. All injections were performed by the same trained investigator to minimize inter-operator variability. Randomization of mice into treatment groups was performed prior to SCI induction, and group allocation remained blinded during injections and subsequent analyses.

Rationale for Dose and Schedule:

The selected dose of 20 µg/kg/day was based on prior literature demonstrating effective modulation of macrophage activity and neuroprotection in murine SCI models, while minimizing systemic side effects. The seven-day consecutive dosing post-injury covers the acute inflammatory phase, which is critical for macrophage-mediated tissue repair and axonal regeneration.

Safety and Ethical Considerations:

All procedures adhered to the Ethics Committee of Nantong First People’s Hospital (Approval No. S20230727-007) guidelines. Mice were housed in specific pathogen-free conditions, with controlled temperature (20–26°C), humidity (40–70%), and a 12-h light/dark cycle, and provided sterile chow and autoclaved water. Injection procedures were performed under strict aseptic conditions, and all disposable materials were safely discarded according to biosafety level 1 standards.

***8. Oil Red O (ORO) Staining***

To evaluate lipid accumulation in spinal cord tissues following injury and treatment, Oil Red O (ORO) staining was performed on frozen spinal cord sections. All steps were carried out under standardized conditions to ensure reproducibility and minimize variability.

Section Preparation:

1. Frozen sections (thickness: 10–15 µm) were retrieved from –80°C storage and allowed to equilibrate to room temperature (20–25°C) for 15–20 minutes to reduce condensation and prevent tissue damage.
2. Sections were fixed in 4% paraformaldehyde (PFA, Servicebio, Wuhan, China) for 15 minutes at room temperature, ensuring crosslinking of proteins and preservation of tissue morphology while maintaining lipid integrity.
3. After fixation, sections were rinsed gently three times with phosphate-buffered saline (PBS, pH 7.4), 5 minutes per rinse, to remove residual fixative.

Preparation of ORO Working Solution:

1. A saturated Oil Red O stock solution was prepared by dissolving ORO powder in isopropanol (100% w/v) at room temperature with gentle agitation until fully dissolved.
2. The working solution was freshly prepared by mixing six parts of saturated ORO stock solution with four parts of distilled water, followed by filtration through a 0.45 µm syringe filter to remove precipitates.
3. The working solution was incubated at 4°C overnight to allow complete dissolution and stabilization of the dye.

Staining Procedure:

1. Sections were immersed in the ORO working solution for 10 minutes at room temperature in the dark to prevent photobleaching and ensure uniform lipid staining.
2. Following staining, background differentiation was performed by sequential immersion in two baths of 60% isopropanol for 3 seconds and 5 seconds, respectively, to remove non-specific staining without extracting lipids.
3. Sections were then briefly rinsed in distilled water twice for 10 seconds each to remove residual isopropanol.

Nuclear Counterstaining:

1. To visualize nuclei, sections were counterstained with hematoxylin for 5 minutes at room temperature.
2. Excess hematoxylin was removed by rinsing in running tap water for 1–2 minutes, followed by brief immersion in distilled water.

Mounting and Imaging:

1. Sections were mounted using neutral balsam to preserve the staining and prevent fading. Care was taken to avoid air bubbles.
2. Lipid droplets appeared as bright red/orange spheres, while nuclei were stained blue.
3. Sections were visualized under a bright-field microscope (Nikon, Tokyo, Japan), and images were captured using a high-resolution digital camera.
4. Quantitative analysis of lipid accumulation was performed using ImageJ software, measuring area, number, and intensity of ORO-positive lipid droplets per field. At least 5 randomly selected fields per section were analyzed to ensure representativeness.

Quality Control and Reproducibility:

1. All staining procedures were performed in parallel for experimental and control groups to minimize batch-to-batch variability.
2. The ORO working solution was freshly prepared or stored at 4°C no longer than 24 hours.
3. All solutions and equipment in contact with tissues were lipid-free and clean to avoid contamination.
4. For reproducibility, all samples were processed by the same operator, and staining intensity was verified on control tissues before analyzing experimental samples.

***9. Hematoxylin and Eosin (HE), Luxol Fast Blue (LFB), and Nissl Staining***

To comprehensively evaluate tissue morphology, myelin integrity, and neuronal structure in spinal cord sections after injury and treatment, HE, LFB, and Nissl staining were performed on paraffin-embedded sections. All staining procedures were standardized to ensure reproducibility and quantitative comparability.

Section Preparation:

1. Paraffin-embedded spinal cord sections (thickness: 5–7 µm) were retrieved from storage and allowed to equilibrate to room temperature (20–25°C) for 15 minutes.
2. Sections were deparaffinized in 2–3 changes of xylene, 5 minutes per change, to completely remove paraffin.
3. Rehydration was performed through a descending ethanol series: 100%, 95%, 80%, and 70%, 3 minutes per step, followed by a rinse in distilled water for 5 minutes to prepare tissue for aqueous staining.

Hematoxylin and Eosin (HE) Staining:

HE staining was used to visualize general tissue morphology, including nuclei, cytoplasm, and extracellular matrix.

1. Sections were immersed in hematoxylin solution (Servicebio) for 10 minutes at room temperature, allowing nuclear DNA to bind the dye and appear blue-purple.
2. Differentiation was performed by briefly immersing the sections in 1% acid alcohol (1% HCl in 70% ethanol) for 30–60 seconds, removing excess hematoxylin to enhance nuclear contrast.
3. Sections were rinsed thoroughly in running tap water for 3–5 minutes to blue the nuclei.
4. Counterstaining was performed by immersing the sections in eosin solution for 3 minutes at room temperature to stain cytoplasm, muscle fibers, and extracellular matrix pink.
5. After staining, sections were rinsed in distilled water, then dehydrated through ascending ethanol concentrations (70%, 80%, 95%, 100%, 3 minutes each).
6. Sections were cleared in xylene for 5 minutes, repeated twice, and mounted using neutral balsam, avoiding air bubbles.

Nissl Staining:

Nissl staining was used to visualize Nissl bodies and neuronal cell bodies, indicating neuronal integrity and injury.

1. Sections were immersed in 0.1% toluidine blue solution (Servicebio) for 15 minutes at room temperature.
2. Excess dye was removed by brief rinsing in distilled water for 10–20 seconds.
3. Nissl bodies appeared as dark blue granules within the cytoplasm, while nuclei were lightly stained.
4. Sections were dehydrated through graded ethanol series (70%, 80%, 95%, 100%), cleared in xylene, and mounted with neutral balsam.
5. Imaging was performed under a bright-field microscope (Nikon, Tokyo, Japan), and quantification of neuronal cell density and Nissl-positive area was performed using ImageJ.

Luxol Fast Blue (LFB) Staining:

LFB staining was used to assess myelin content and integrity in the spinal cord.

1. Myelin Staining Solution A (Servicebio) was preheated to 60°C for 30 minutes. Sections were then immersed in the preheated solution and stained for 1 hour at 60°C.
2. Differentiation steps were performed to remove non-specific staining:
   - Sections were immersed briefly (2 seconds) in warm Myelin Staining Solution B.
   - Followed by immersion in Myelin Staining Solution C for 15 seconds.
3. Sections were dried at 65°C for 30 minutes, then cooled to room temperature.
4. Counterstaining was performed with eosin solution, followed by dehydration through graded ethanol, clearing in xylene, and mounting with neutral balsam.
5. Myelin appeared as deep blue, nuclei as red/purple, allowing assessment of demyelination. Quantitative analysis included myelin area fraction and intensity using ImageJ.

Quality Control and Reproducibility:

1. All staining steps were performed in parallel for experimental and control groups to minimize batch effects.
2. Fresh reagents were prepared and filtered to avoid precipitates.
3. Each batch of slides included internal positive and negative controls (normal spinal cord sections).
4. All imaging was performed using identical microscope settings, and quantitative analyses were performed blind to experimental groups.

***10. Immunofluorescence (IF) Staining***

Immunofluorescence staining was performed to visualize specific protein expression and subcellular localization in cultured cells and spinal cord tissue sections. Detailed procedures were standardized to ensure reproducibility and quantitative reliability.

Sample Preparation:

Cultured cells:

1. Cells were washed gently with phosphate-buffered saline (PBS, pH 7.4) to remove culture medium.
2. Fixed with 4% paraformaldehyde (PFA) for 15 minutes at room temperature (20–25°C) to preserve cellular morphology and antigenicity.
3. Cells were washed 3 times with PBS (5 minutes each) to remove residual fixative.

Paraffin-embedded tissue sections:

1. Sections (5–7 µm thick) were deparaffinized in 2–3 changes of xylene, 5 minutes each.
2. Rehydrated through a graded ethanol series: 100%, 95%, 80%, 70%, 3 minutes each, followed by a rinse in distilled water for 5 minutes.
3. Heat-induced antigen retrieval was performed by incubating sections in sodium citrate buffer (10 mM, pH 6.0, Beyotime) at 95–100°C for 15–20 minutes using a water bath or pressure cooker.
4. Sections were allowed to cool to room temperature for 30 minutes, followed by three washes in PBS.

Quenching and Blocking:

1. Endogenous peroxidase and autofluorescence were quenched by incubating the sections with peroxidase blocking solution (Beyotime) for 30 minutes at room temperature, then washed 3 times with PBS (5 minutes each).
2. Non-specific binding sites were blocked with blocking buffer (5% BSA or 10% normal goat serum in PBS, Beyotime) for 1 hour at room temperature.
3. For cultured cells, blocking buffer was applied directly after fixation and permeabilization (0.1% Triton X-100 in PBS for 10 minutes at room temperature) if intracellular proteins were targeted.

Primary Antibody Incubation:

1. Primary antibodies were diluted in blocking buffer at optimized concentrations (typically 1:100–1:500, depending on antibody).
2. Samples were incubated overnight at 4°C in a humidified chamber to prevent drying.
3. After incubation, sections/cells were washed three times with PBS (5 minutes each) to remove unbound antibody.

Secondary Antibody Incubation:

1. Appropriate fluorophore-conjugated secondary antibodies (Alexa Fluor 488, 555, or 647, Invitrogen or Beyotime) were diluted in blocking buffer (typically 1:500).
2. Samples were incubated for 1 hour at room temperature in the dark to avoid photobleaching.
3. Sections/cells were washed three times with PBS (5 minutes each) after secondary antibody incubation.

Nuclear Counterstaining and Mounting:

1. Nuclear staining was performed using DAPI (4′,6-diamidino-2-phenylindole, 1 µg/mL) for 10 minutes at room temperature in the dark.
2. Excess DAPI was removed by rinsing 3 times with PBS.
3. Samples were mounted with anti-fade mounting medium (Beyotime) to preserve fluorescence signals and prevent photobleaching.
4. Coverslips were sealed with clear nail polish or appropriate sealant to prevent drying.

Imaging and Quantitative Analysis:

1. Imaging was performed using a fluorescence microscope (Nikon, Tokyo, Japan) or confocal microscope, with identical exposure settings across experimental groups to ensure comparability.
2. Fluorescence intensity, area, or colocalization was quantified using ImageJ/Fiji or similar software.
3. At least 3–5 fields per sample were captured, and analyses were performed blind to experimental groups.

Quality Control:

1. Negative controls: Samples were incubated with secondary antibody only to monitor background fluorescence.
2. Positive controls: Sections known to express target proteins were included to confirm antibody specificity.
3. All reagents were freshly prepared, and light exposure was minimized throughout to reduce photobleaching.
4. Experiments were repeated at least three times to confirm reproducibility.

***11. Myelin Debris (MD) Preparation***

Purpose: To isolate pure myelin debris (MD) from mouse brain tissue for in vitro macrophage phagocytosis experiments.

Animal Handling and Tissue Collection:

1. Experimental animals: C57BL/6 mice, aged 8–12 weeks, were used.
2. Euthanasia: Mice were euthanized by cervical dislocation, followed by immersion in 75% ethanol for 15 minutes to disinfect the fur and skin surface.
3. Decapitation and brain removal: Brains were carefully excised using sterile surgical scissors and forceps, avoiding mechanical damage.
4. Initial washing: Excised brains were transferred into a pre-chilled 10-cm sterile culture dish containing 10 mL ice-cold Tris·Cl buffer (prepared by combining 480 mL ddH₂O, 10 mL 1 M Tris·Cl, and 10 mL 0.5 M Na₂EDTA), and gently rinsed to remove blood and residual surface debris.
5. Tissue transfer: Cleaned brain tissue was placed into a 50-mL sterile centrifuge tube on ice to maintain low temperatures and preserve myelin integrity.

Tissue Homogenization and Sucrose Gradient Preparation:

Homogenization:

1. Added 0.32 M sucrose solution to the 50-mL tube to a final volume of 25 mL.
2. Tissue was homogenized using a Dounce homogenizer for 2 minutes on ice, ensuring complete disruption of tissue while minimizing mechanical damage to myelin membranes.
3. The homogenate was kept on ice at all times to prevent protein degradation and lipid oxidation.

Sucrose density-gradient setup:

1. Prepared 0.83 M sucrose solution and aliquoted 3 mL into eight ultracentrifuge tubes.
2. Homogenate was gently layered on top of the sucrose solution using a pipette, taking care not to mix the layers, which is critical for effective separation of myelin debris.
3. Tubes were balanced precisely on an analytical scale before ultracentrifugation.

Ultracentrifugation and Myelin Debris Collection:

First centrifugation:

1. Ultracentrifuged at 100,000 × g for 45 minutes at 4°C.
2. After centrifugation, a white myelin debris band appeared at the sucrose interface.
3. This band was carefully collected with a pipette, avoiding contamination from other layers.
4. Collected debris was resuspended in 2 mL ice-cold Tris·Cl buffer and homogenized gently.

Second centrifugation (purification step):

1. Resuspended myelin debris was distributed into four ultracentrifuge tubes (5 mL per tube).
2. Centrifuged again at 100,000 × g for 45 minutes at 4°C.
3. The resulting pellets were resuspended in 2 mL Tris·Cl buffer and pooled into two tubes.

Final centrifugation:

1. To remove residual sucrose and small debris, samples were centrifuged at 22,000 × g for 10 minutes at 4°C.
2. Supernatant was discarded, and the final myelin pellet was collected.

Myelin Debris Quantification and Storage:

1. The myelin pellet was weighed using a high-precision analytical balance.
2. Resuspended in sterile phosphate-buffered saline (PBS) to a final concentration of 100 mg/mL, ensuring consistent dosing for subsequent experiments.
3. Aliquoted into sterile, nuclease-free microcentrifuge tubes to avoid repeated freeze–thaw cycles.
4. Stored at –80°C for up to six months. For long-term storage, samples were thawed on ice and kept on ice during handling.

Quality Control:

1. Visual inspection: Myelin debris should appear as a homogeneous white suspension.
2. Purity assessment: Random aliquots were examined under a light microscope to confirm the presence of myelin fragments and absence of large cellular debris.
3. Sterility: All steps were performed under sterile conditions to avoid bacterial contamination.
4. Reproducibility: The protocol was repeated with 3–5 independent mouse brains to ensure consistency in yield and quality.

***12. Extraction, Culture, and Treatment of Mouse Bone Marrow-Derived Macrophages (BMDMs)***

Purpose: To isolate, differentiate, and functionally manipulate mouse BMDMs for in vitro assays, including myelin debris (MD) uptake and pharmacological treatments.

Animal Handling and Bone Marrow Collection:

1. Experimental animals: Male or female C57BL/6 mice, 6–8 weeks old, were used.
2. Euthanasia and sterilization: Mice were anesthetized with isoflurane or an approved deep anesthesia protocol, followed by immersion in 75% ethanol for 5–10 minutes to disinfect the skin and reduce microbial contamination.
3. Bone isolation: Femurs and tibias were carefully dissected using sterile surgical scissors and forceps. Muscles, ligaments, and connective tissues were completely removed under a sterile biosafety cabinet.
4. Secondary disinfection: Bones were briefly rinsed again with 75% ethanol to maintain sterility.
5. Preparation for flushing: Both ends of the bones were cut with sterile scissors to expose the bone marrow cavity.

Bone Marrow Flushing and Single-Cell Suspension Preparation:

Marrow flushing:

1. Using a sterile 5-mL syringe and 25G needle, 5 mL of pre-warmed DMEM (KeyGEN, Nanjing, China) was injected into the marrow cavity to flush out bone marrow cells into a 50-mL sterile tube.
2. The flushing was repeated two additional times to maximize recovery.

Cell straining: The resulting cell suspension was passed through a 70-µm sterile cell strainer to remove bone fragments and connective tissue debris.

Red blood cell (RBC) lysis:

1. Cells were resuspended in red blood cell lysis buffer (Beyotime, China) and incubated for 3 minutes at room temperature with gentle inversion.
2. Following lysis, the suspension was centrifuged at 1,300 rpm for 5 minutes at 4°C, and the supernatant containing lysed erythrocytes was discarded.

BMDM Culture and Differentiation:

1. Seeding: The cell pellet was resuspended in DMEM supplemented with 5% heat-inactivated fetal bovine serum (FBS; Gibco, Grand Island, NY, USA) and 30 ng/mL recombinant mouse macrophage colony-stimulating factor (M-CSF; HY-P70263A, MedChemExpress, USA).
2. Initial culture: Cells were seeded in non-tissue culture-treated 6-well plates at a density of approximately 1 × 10⁶ cells/well and incubated at 37°C in a humidified 5% CO₂ incubator for 3 days.
3. Medium change: After 3 days, half of the medium was gently replaced with fresh M-CSF-containing DMEM to remove non-adherent cells while retaining differentiating macrophages.
4. Differentiation: Cells were continuously cultured for an additional 5–7 days, with medium replaced every 2–3 days, until adherent cells exhibited typical macrophage morphology (large, spread, and polygonal with ruffled membranes).

Functional Treatment of BMDMs:

Pharmacological pretreatment: Differentiated BMDMs were treated under sterile conditions with:

1. Pifithrin-β (PFT-β, 10 μM, 24 h) – a selective p53 inhibitor.
2. Exendin-4 (Ex-4, 100, 200, or 400 nM, 24 h) – a GLP-1 receptor agonist.
3. Compound 5d (1 μM, 24 h) – experimental small molecule.
4. Recombinant mouse Gas6 (rGas6, 500 ng/mL, 2 h) – to simulate Gas6 signaling.
5. BML-275 (1 μM, 24 h) – AMPK inhibitor.

All treatments were prepared in complete DMEM containing 5% FBS and administered under standard culture conditions (37°C, 5% CO₂).

1. Myelin debris stimulation: Following pharmacological pretreatment, BMDMs were stimulated with MD at a final concentration of 1 mg/mL for 12 hours to assess uptake, efferocytosis, and downstream signaling responses.
2. Controls: Untreated BMDMs and vehicle-treated groups were included as negative controls to distinguish baseline effects from pharmacological or MD-induced changes.

Quality Control and Morphological Verification:

1. Purity assessment: BMDMs were characterized by F4/80 and CD11b immunostaining prior to functional assays. Purity >90% was considered acceptable.
2. Morphology check: Adherent cells were inspected using a phase-contrast microscope to verify macrophage differentiation (large, spread cytoplasm with ruffled edges).
3. Viability: Cell viability was confirmed by Trypan Blue exclusion assay before treatments.
4. Reproducibility: The entire procedure was repeated with 3–5 independent mice per experiment, and BMDM yield, morphology, and differentiation efficiency were recorded.

***13. Senescence-associated β-galactosidase (SA-β-gal) Staining***

Purpose: To detect cellular senescence in BMDMs by visualizing β-galactosidase activity at pH 6.0, indicated by the appearance of blue-stained cytoplasmic granules.

Materials and Preparation:

1. Reagents and kit: β-Galactosidase Staining Kit (C0602, Beyotime, China) was used, including fixative solution, SA-β-gal staining solution, and washing buffers.
2. Cell culture: BMDMs were seeded in 6-well plates at a density of approximately 1 × 10⁵–2 × 10⁵ cells/well and treated according to experimental design (e.g., MD stimulation, pharmacological pretreatment). Cells were at 70–80% confluency prior to staining to avoid overgrowth and ensure uniform staining.
3. Staining solution preparation: SA-β-gal staining solution was freshly prepared immediately before use, according to the kit instructions, and pre-warmed to room temperature.

Fixation of Cells:

1. Cells were gently washed once with 1 mL PBS per well to remove residual culture medium and serum components that could interfere with staining.
2. Fixation: 1 mL of fixative solution from the kit was added per well. Cells were incubated at room temperature for 15 minutes.
   - Care was taken to avoid detaching adherent macrophages by gently adding and removing solutions along the well edge.
3. After fixation, cells were rinsed three times with PBS, 5 minutes each, to remove residual fixative and prevent background staining.

SA-β-gal Staining:

1. Addition of staining solution: 1 mL of freshly prepared SA-β-gal staining solution was added to each well, ensuring the entire monolayer was covered.
2. Incubation: Plates were incubated at 37°C in a dry incubator without CO₂ to maintain pH 6.0, which is critical for SA-β-gal specificity.
3. Duration: Cells were incubated overnight (~16–18 hours).

Staining time was carefully monitored; excessive incubation (>24 h) may increase background.

1. Observation: After incubation, cells were gently washed once with PBS to remove excess staining solution.

Microscopy and Imaging:

1. Cells were examined under a bright-field light microscope (Nikon, Tokyo, Japan) using a 10× or 20× objective.
2. Identification of senescent cells: Senescent BMDMs were identified by the presence of blue cytoplasmic granules. Non-senescent cells appeared colorless or lightly stained.
3. Image acquisition: Representative fields (at least five random fields per well) were photographed for quantification.

Quantification and Quality Control:

1. Quantification: The percentage of SA-β-gal-positive cells was calculated by dividing the number of blue-stained cells by the total number of cells in each field.
2. Replicates: Experiments were performed in triplicate wells per condition and repeated with BMDMs from at least three independent mice.
3. Controls: Untreated BMDMs served as negative controls, while known senescence-inducing conditions (e.g., H₂O₂ treatment, if applicable) served as positive controls.
4. Notes: Care was taken to avoid excessive light exposure during and after staining, as prolonged exposure may bleach the blue signal.

***14. Primary Astrocyte and Neuron Isolation***

Primary Astrocyte Isolation:

Purpose: To obtain highly purified cortical astrocytes from neonatal mice for in vitro experiments.

1. Animals: Cerebral cortices were collected from postnatal day 1–3 (P1–P3) C57BL/6J mice, euthanized according to institutional animal care guidelines.
2. Tissue dissection: Brains were rapidly removed and transferred to ice-cold HBSS without calcium and magnesium. Cortical tissues were dissected free of meninges under a stereomicroscope and minced into ~2×2 mm fragments using sterile scalpel blades.
3. Centrifugation: Tissue fragments were centrifuged at 800 rpm for 3 minutes at 4°C to pellet the tissue and remove debris.
4. Enzymatic digestion: Pellets were incubated with 0.25% trypsin (Gibco, USA) at 37 °C for 5 minutes with gentle agitation every minute to dissociate cells.
5. Termination of digestion: The enzymatic reaction was stopped by adding DMEM supplemented with 10% FBS (Gibco).
6. Mechanical dissociation: Tissue suspension was gently triturated using a flame-polished Pasteur pipette until a single-cell suspension was achieved.
7. Filtration and centrifugation: The suspension was filtered through a 70 μm sterile cell strainer to remove undigested debris, then centrifuged at 1300 rpm for 5 minutes at 4 °C.
8. Seeding and culture: The cell pellet was resuspended in DMEM + 10% FBS and plated into poly-D-lysine (PDL, 50 μg/mL)-coated T25 flasks. Cells were maintained at 37 °C in a humidified incubator with 5% CO₂.
9. Medium change: After 24 h, non-adherent cells were removed by replacing the medium, followed by medium changes every 3 days.
10. Microglia removal: After 14–20 days, flasks were shaken at 200 rpm for 3–4 h at 37 °C to remove loosely attached microglia. For further astrocyte purification, an additional 12-hour shaking step at 200 rpm was performed.
11. Trypsinization for downstream use: Purified astrocytes were harvested using 0.05% trypsin-EDTA for functional assays, immunocytochemistry, or co-culture experiments.
12. Quality control: Astrocyte purity was confirmed via GFAP immunostaining (>95% GFAP⁺ cells).

Primary Cortical Neuron Isolation:

Purpose: To obtain viable cortical neurons for in vitro studies, including co-culture and functional assays.

1. Animals: Cortical neurons were obtained from embryonic day 16–18 (E16–E18) C57BL/6J mouse embryos.
2. Tissue dissection: Embryos were removed from the uterus and placed on ice in HBSS without calcium and magnesium. Cerebral cortices were dissected, meninges removed, and tissue minced into small fragments.
3. Enzymatic digestion: Tissue fragments were incubated in 2 mg/mL papain (Sigma-Aldrich) in DMEM at 37 °C for 20–30 minutes, with gentle swirling every 5 minutes to ensure uniform digestion.
4. Termination of digestion: Enzymatic activity was halted by adding DMEM containing 2% horse serum (Gibco).
5. Mechanical dissociation: The tissue was gently triturated on ice using a fire-polished Pasteur pipette until a single-cell suspension was obtained.
6. Filtration and centrifugation: The suspension was passed through a 70 μm sterile cell strainer and centrifuged at 1000 rpm for 5 minutes at 4 °C.
7. Seeding and culture: The cell pellet was resuspended in DMEM + 10% FBS and plated onto PDL-coated 6-well plates at a density of 7 × 10⁵ cells/well.
8. Initial attachment: Cells were allowed to attach for 4 hours at 37 °C, 5% CO₂, after which the medium was replaced with Neurobasal medium supplemented with 1× GlutaMAX and 2% B27 supplement (Gibco) to support neuronal survival and reduce glial proliferation.
9. Medium maintenance: Half of the medium was replaced every two days, with care to minimize mechanical disturbance to neurons.
10. Quality control: Neuronal purity and health were assessed via MAP2 or βIII-tubulin immunostaining (>90% neurons), and morphological features (neurite outgrowth, absence of debris) were monitored.

Notes / Critical Points:

1. All dissection and digestion steps were performed under sterile conditions in a biosafety cabinet to avoid contamination.
2. Temperature and timing were strictly controlled to maximize cell viability.
3. Poly-D-lysine coating was used to enhance cell adhesion.
4. Shaking steps for astrocytes must be optimized to remove microglia without detaching astrocytes.
5. All reagents were pre-warmed or pre-chilled as appropriate to minimize stress on primary cells.

***15. Preparation and Labeling of Apoptotic Neurons***

Purpose: To generate fluorescently labeled apoptotic cortical neurons suitable for in vitro efferocytosis assays with BMDMs.

1. Isolation of primary cortical neurons: Primary cortical neurons were prepared from embryonic day 16–18 (E16–E18) C57BL/6J mouse embryos following the protocol described in Section 2.1114. Neurons were plated onto poly-D-lysine (50 μg/mL)-coated culture plates at a density of 7 × 10⁵ cells/well and maintained in Neurobasal medium supplemented with 1× GlutaMAX and 2% B27. Medium was half-changed every 2 days.
2. Neuronal culture before apoptosis induction: Neurons were cultured for 7 days in vitro (DIV7) to allow for neurite outgrowth and synaptic network formation, which ensures physiological relevance in efferocytosis assays.
3. Induction of apoptosis: Neuronal apoptosis was triggered by adding 1 μM staurosporine (Sigma-Aldrich, USA) directly to the culture medium and incubating the cells at 37 °C in a 5% CO₂ incubator for 4 hours. Care was taken to ensure uniform distribution of staurosporine by gently swirling the plates every 30 minutes.
4. Washing and resuspension: After apoptosis induction, neurons were carefully washed twice with sterile PBS to remove residual staurosporine and dead cell debris. Cells were then gently resuspended in serum-free DMEM to maintain viability and prevent interference from serum proteins during subsequent labeling.
5. Fluorescent labeling of apoptotic neurons: To visualize apoptotic neurons during efferocytosis, cells were incubated with 5 μM carboxyfluorescein diacetate succinimidyl ester (CFSE; MedChemExpress, USA) for 15 minutes at 37 °C in the dark. CFSE is a cell-permeable dye that covalently binds intracellular proteins, allowing long-lasting fluorescence labeling.
6. Removal of excess dye: Following CFSE incubation, neurons were washed three times with sterile PBS to remove unbound dye. Cells were resuspended in fresh serum-free DMEM at an appropriate concentration for co-culture.
7. Co-culture with BMDMs: Labeled apoptotic neurons were added to bone marrow-derived macrophages (BMDMs) at a ratio of approximately 5:1 (neurons:macrophages) in culture medium. Co-cultures were incubated at 37 °C with 5% CO₂ for the desired time points (e.g., 1–4 hours) to allow macrophage engulfment of apoptotic neurons.
8. Assessment of efferocytosis:

Live-cell imaging: Engulfment of CFSE-labeled apoptotic neurons by macrophages was monitored using a fluorescence microscope (Nikon, Tokyo, Japan). Images were captured at multiple time points to quantify the efficiency of efferocytosis.

Immunofluorescence staining (optional): After co-culture, cells were fixed with 4% paraformaldehyde for 15 minutes, permeabilized, and stained with lysosomal markers (e.g., LAMP1) to visualize the digestion of apoptotic neurons within macrophage lysosomes.

1. Quality control:

Apoptotic induction efficiency was verified by Annexin V/PI staining, ensuring >70% of neurons exhibited early apoptotic features.

CFSE labeling efficiency and uniformity were checked by fluorescence microscopy before co-culture.

Macrophage viability was monitored during co-culture to avoid artifacts due to cell death.

Critical Points / Notes:

1. All steps involving CFSE were performed in the dark to prevent photobleaching.
2. Gentle pipetting was used throughout to avoid mechanical damage to fragile apoptotic neurons.
3. Serum-free conditions during labeling and co-culture help reduce background fluorescence and non-specific binding.
4. Time points and neuron-to-macrophage ratios can be optimized depending on experimental goals.

***16. Indirect Co-culture of BMDMs with Primary Astrocytes and Neurons***

Purpose: To assess the paracrine effects of treated BMDMs on primary astrocytes and neurons, including astrocytic scar formation, neuronal axonal growth, and apoptosis.

Collection of macrophage-conditioned medium (MCM):

1. After completion of experimental treatments (e.g., MD stimulation, Ex-4, rGas6, PFT-β, or other pharmacological interventions) on BMDMs, culture supernatants were carefully collected to avoid disturbing adherent macrophages.
2. Supernatants were centrifuged at 300 × g for 5 minutes at 4 °C to remove detached cells and large debris.
3. The clarified supernatant was then filtered through a 0.22 μm sterile membrane filter to remove residual cellular debris and microorganisms, yielding macrophage-conditioned medium (MCM) suitable for indirect co-culture.
4. MCM was stored on ice and used immediately or aliquoted and stored at –80 °C for no longer than 1 month to preserve bioactivity.

Preparation of primary astrocytes and neurons:

1. Primary astrocytes and cortical neurons were isolated and cultured as described in Section 2.1114.
2. Astrocytes were seeded onto poly-D-lysine-coated plates or coverslips at a density of 2 × 10⁵ cells/well in a 24-well plate and cultured until 80–90% confluent.
3. Cortical neurons were plated on PDL-coated 6-well plates or coverslips at 7 × 10⁵ cells/well and cultured for 7–10 days in vitro before MCM treatment.

Application of MCM to astrocytes and neurons:

1. The medium of astrocytes or neurons was replaced with 50–100% MCM, diluted with fresh DMEM (astrocytes) or Neurobasal medium with B27 and GlutaMAX (neurons) as needed.
2. Cells were incubated with MCM for 24 hours at 37 °C in a 5% CO₂ humidified incubator to allow the paracrine factors secreted by BMDMs to exert their biological effects.

Experimental endpoints and assessments:

1. Astrocytic scar formation: Immunofluorescence staining for GFAP and vimentin was performed to evaluate astrocyte activation and morphological changes. The area and intensity of GFAP-positive processes were quantified using image analysis software.
2. Neuronal axonal growth: Immunostaining for βIII-tubulin (Tuj1) or MAP2 was conducted to visualize neuronal processes. Axon length, branching, and density were measured to assess neurotrophic or inhibitory effects.
3. Neuronal apoptosis: TUNEL staining or Annexin V/PI assays were applied to detect apoptotic neurons. The percentage of TUNEL-positive neurons or apoptotic index was quantified.
4. Optional assays include qPCR or ELISA to detect cytokine or growth factor levels in astrocytes or neurons after MCM treatment.

Quality control and critical points:

1. MCM should be freshly prepared or thawed carefully to avoid degradation of secreted factors.
2. Supernatants should be handled gently to prevent contamination with residual macrophages, which could confound results.
3. All steps involving neurons should minimize mechanical disturbance to preserve neurite integrity.
4. Experiments should include control groups treated with unconditioned medium or MCM from untreated BMDMs to distinguish specific paracrine effects.

***17. Flow Cytometry (FCM) for Neuronal Apoptosis***

Purpose: To quantitatively assess apoptosis in primary cortical neurons following treatment with macrophage-conditioned medium (MCM) or other experimental interventions.

Cell harvest and preparation:

1. Primary neurons were gently dissociated from culture plates using 0.05% trypsin-EDTA (Gibco, USA) for 2–3 minutes at 37 °C.
2. Enzymatic digestion was immediately terminated by adding 2 volumes of DMEM containing 10% FBS to prevent over-digestion.
3. Cells were collected by centrifugation at 1,000 rpm for 5 minutes at 4 °C, and the supernatant was carefully discarded.
4. The cell pellet was washed twice with cold PBS to remove residual serum and enzymes.

Staining for apoptosis:

1. Cells were resuspended in 100 µL of 1× Binding Buffer (provided in the Annexin V-FITC/PI Apoptosis Detection Kit, YFXCA03, YIFEIXUE BioTech) to achieve a density of ~1 × 10⁶ cells/mL.
2. Annexin V-FITC (5 µL) was added to label phosphatidylserine externalization, and Propidium Iodide (PI) (5 µL) was added to label membrane-compromised late apoptotic or necrotic cells.
3. The mixture was gently vortexed and incubated for 15 minutes at room temperature in the dark to prevent photobleaching.

Flow cytometry acquisition:

1. After staining, 400 µL of 1× Binding Buffer was added to each sample to reach a final volume of 500 µL.
2. Samples were immediately analyzed using a FACSVerse 8 flow cytometer (BD Biosciences, NJ, USA) equipped with a 488 nm laser.
3. Compensation controls were prepared using unstained cells, Annexin V-only stained cells, and PI-only stained cells to correctly set gates and compensate for spectral overlap.

Data analysis:

1. Data were acquired for 10,000–20,000 events per sample to ensure statistical reliability.
2. Neuronal populations were gated based on forward scatter (FSC) and side scatter (SSC) to exclude debris.
3. Apoptotic neurons were categorized as follows:
   - 1. Early apoptosis: Annexin V-FITC⁺ / PI⁻
     2. Late apoptosis/necrosis: Annexin V-FITC⁺ / PI⁺
     3. Live cells: Annexin V-FITC⁻ / PI⁻
4. Quantitative analysis was performed using FlowJo v10 software (BD Biosciences), and apoptosis rates were expressed as percentages of total neuronal events.

Critical considerations:

1. All steps involving neurons were performed gently to minimize mechanical stress and false-positive apoptosis signals.
2. Staining and analysis were conducted promptly after harvesting to prevent apoptosis induced by prolonged handling.
3. Biological replicates (n ≥ 3) were used for each experimental group, and unstained and single-stained controls were included in each run to ensure accuracy.

***18. Lentivirus (LV) Transfection of BMDMs***

Purpose: To achieve stable knockdown of Gas6 in bone marrow-derived macrophages (BMDMs) using lentiviral-mediated RNA interference.

Cell preparation:

1. BMDMs were seeded in 6-well plates at a density of 2×10⁵ cells/well 24 hours prior to transduction to achieve 60–70% confluence at the time of infection.
2. Cells were maintained in DMEM supplemented with 5% FBS and 30 ng/mL M-CSF at 37 °C in a humidified incubator with 5% CO₂.
3. Prior to transduction, the culture medium was replaced with fresh growth medium to remove dead cells and debris.

Lentivirus preparation and infection:

1. Lentiviral vectors carrying either scrambled shRNA (LV-NC) or shRNA targeting Gas6 (LV-shGas6) at a titer of 1×10⁸ TU/mL were thawed on ice and gently mixed by pipetting.
2. To enhance transduction efficiency, HitransG A transduction enhancer (1×; GeneChem, Shanghai, China) was added to the viral inoculum according to the manufacturer’s instructions.
3. BMDMs were exposed to the viral mixture by adding 500 µL of virus-containing medium per well, ensuring even distribution across the culture surface.

Incubation and medium replacement:

1. Cells were incubated at 37 °C with 5% CO₂ for 12 hours, with gentle swirling every 3–4 hours to maintain uniform contact between virus and cells.
2. After 12 hours, the viral-containing medium was carefully removed to minimize cytotoxicity.
3. Cells were washed once with pre-warmed PBS and replaced with 2 mL of complete growth medium (DMEM + 5% FBS + 30 ng/mL M-CSF) per well.

Post-transduction culture and verification:

1. Transduced BMDMs were cultured for an additional 60 hours to allow sufficient expression of shRNA and knockdown of Gas6.
2. Morphology was monitored under a light microscope to assess cell health and adherence.
3. Knockdown efficiency was confirmed by qRT-PCR and/or Western blot analysis prior to functional assays.

Critical considerations:

1. All steps were performed under sterile conditions in a biosafety cabinet to prevent contamination.
2. The multiplicity of infection (MOI) was optimized to balance transduction efficiency and cell viability.
3. Lentivirus exposure time was strictly limited to 12 hours to reduce cytotoxic effects on BMDMs.
4. Biological replicates (n ≥ 3) were included for reproducibility, and untransduced cells were used as additional controls.

***19. Panoramic Imaging for Label-Free Live Cells Using an Optical Diffraction Tomography (ODT) Microscope***

Purpose: To capture high-resolution, label-free, three-dimensional (3D) images of live BMDMs under different experimental conditions, enabling quantitative and qualitative analysis of cell morphology, organelle dynamics, and cellular interactions.

Cell preparation:

1. BMDMs were seeded on glass-bottom dishes (MatTek, USA) at a density of 1×10⁵ cells/dish 24 hours before imaging to allow for adequate adhesion.
2. Cells were maintained in DMEM supplemented with 5% FBS and 30 ng/mL M-CSF and incubated at 37 °C with 5% CO₂ until the time of imaging.
3. Prior to imaging, culture medium was replaced with phenol red-free DMEM to reduce background optical interference.

ODT microscope setup:

1. Label-free imaging was performed using a live-cell super-resolution panoramic microscope (MH-Holiview, Cheng Guan Optics Technology Co., Ltd., Nantong, China), integrated with a commercial inverted microscope (IX83, Olympus, Japan).
2. The system employs an off-axis holographic optical diffraction tomography (ODT) module with a galvo-mirror scanning mechanism, enabling rapid acquisition of multi-angle phase images for 3D reconstruction.
3. The microscope was equipped with a temperature-controlled chamber (37 °C) and humidified CO₂ environment (5%) to maintain physiological conditions throughout imaging.
4. Fluorescence excitation modules were available for optional dual-channel imaging, although label-free mode was used for this study.
5. Image acquisition and processing were controlled via custom MATLAB 2021a software, capable of stitching multiple fields of view for panoramic visualization.

Imaging parameters and acquisition:

1. Individual BMDMs were imaged at high spatiotemporal resolution, with a voxel size of 0.1 μm × 0.1 μm × 0.2 μm and a temporal resolution of 1 frame per minute for dynamic studies.
2. For panoramic imaging, multiple adjacent fields of view (FOVs) were captured and computationally stitched to obtain a large-scale overview of cellular populations and their interactions.
3. Each experiment included at least three independent dishes per condition, with 10–15 representative cells analyzed per dish.

Post-processing and analysis:

1. Raw ODT holograms were reconstructed into 3D refractive index (RI) tomograms using the microscope’s proprietary reconstruction algorithms.
2. Morphological features (cell volume, sphericity, membrane protrusions) and intracellular structures (nuclear and organelle distributions) were quantified.
3. Data were exported for statistical analysis using ImageJ (NIH, USA) and MATLAB.

Critical considerations:

1. All imaging was performed under sterile conditions to avoid contamination.
2. Cells were imaged without fixation or labeling to preserve physiological integrity and allow real-time observation of cellular dynamics.
3. Light exposure and laser power were kept at minimal levels to prevent phototoxicity during prolonged imaging.
4. Calibration of the ODT system was performed before each experiment to ensure accurate refractive index measurements and spatial resolution.

***20. Western Blot (WB)***

Purpose: To detect and quantify specific proteins in BMDMs under different experimental conditions, allowing assessment of protein expression levels and post-translational modifications.

Protein extraction:

1. BMDMs were harvested and washed twice with cold phosphate-buffered saline (PBS) to remove residual medium.
2. Cells were lysed on ice using RIPA lysis buffer (KeyGEN, Nanjing, China) supplemented with protease inhibitor cocktail (e.g., PMSF, 1 mM) and phosphatase inhibitor cocktail to preserve protein integrity and phosphorylation status.
3. Lysates were incubated on ice for 30 minutes with occasional vortexing and then centrifuged at 12,000 × g for 15 minutes at 4 °C to remove cell debris.
4. The supernatant was collected, and protein concentration was determined using the bicinchoninic acid (BCA) protein assay kit (KeyGEN) according to the manufacturer’s instructions.

SDS-PAGE and transfer:

1. Equal amounts of protein (typically 20–40 μg per lane) were mixed with 4× Laemmli sample buffer containing 5% β-mercaptoethanol, and boiled at 95 °C for 5 minutes for denaturation.
2. Proteins were separated on 10–12% SDS-polyacrylamide gels according to molecular weight. Electrophoresis was performed at 80 V for stacking gel and 120 V for resolving gel until the dye front reached the bottom of the gel.
3. Proteins were transferred onto polyvinylidene fluoride (PVDF) membranes (0.45 μm pore size) using a wet transfer system at 100 V for 90 minutes at 4 °C. Membranes were pre-activated with 100% methanol for 1 minute before transfer.

Blocking and antibody incubation:

1. Membranes were blocked with 5% non-fat milk in Tris-buffered saline with 0.1% Tween-20 (TBST) for 1 hour at room temperature to prevent non-specific binding.
2. Membranes were incubated overnight at 4 °C with primary antibodies diluted in TBST containing 1% BSA (antibody concentrations as listed in Table S1).
3. After washing three times with TBST (10 minutes each), membranes were incubated with HRP-conjugated secondary antibodies for 1 hour at room temperature in the dark.
4. Detection and quantification:
5. Protein bands were visualized using enhanced chemiluminescence (ECL) substrate and captured with an imaging system (Syngene, Cambridge, UK).
6. Band intensity was quantified using ImageJ software (NIH, Bethesda, MD, USA). Relative protein expression was normalized to housekeeping proteins (e.g., GAPDH or β-actin).
7. All experiments were performed in triplicate, and representative blots are shown.

Critical considerations:

1. Ensure equal protein loading by quantifying protein concentration accurately and confirming uniform housekeeping protein levels.
2. Maintain low temperatures during lysis, centrifugation, and transfer to preserve protein integrity.
3. Avoid prolonged exposure to light during ECL detection to prevent signal saturation.

***21. RNA Isolation and Real-Time Quantitative PCR (qPCR)***

Purpose: To quantify mRNA expression levels of target genes in BMDMs, neurons, or astrocytes under different experimental conditions.

RNA isolation:

1. Cells were harvested and washed twice with cold phosphate-buffered saline (PBS) to remove residual culture medium.
2. Total RNA was extracted using TRIzol reagent (YIFEIXUE, Nanjing, China) according to the manufacturer’s protocol:
3. Cells were lysed directly in the culture dish by adding 1 mL of TRIzol per well of a 6-well plate.
4. Lysates were transferred to RNase-free microcentrifuge tubes and incubated for 5 minutes at room temperature to ensure complete dissociation of nucleoprotein complexes.
5. 0.2 mL of chloroform was added per 1 mL of TRIzol, vigorously shaken for 15 seconds, and incubated for 3 minutes at room temperature.
6. Samples were centrifuged at 12,000 × g for 15 minutes at 4 °C. The upper aqueous phase containing RNA was carefully transferred to a new RNase-free tube.
7. RNA was precipitated by adding 0.5 mL of isopropanol per 1 mL of TRIzol used, incubated at room temperature for 10 minutes, and centrifuged at 12,000 × g for 10 minutes at 4 °C.
8. The RNA pellet was washed with 75% ethanol, air-dried briefly, and resuspended in RNase-free water.

RNA quantification and quality assessment:

1. RNA concentration and purity were measured using a spectrophotometer (NanoDrop or equivalent).
2. Purity was assessed by the A260/A280 ratio, with values between 1.8–2.0 considered acceptable. RNA integrity was optionally verified by electrophoresis on a 1% agarose gel.

cDNA synthesis (reverse transcription):

1. 1 µg of total RNA was reverse-transcribed into cDNA using a reverse transcription kit (YIFEIXUE, Nanjing, China).
2. The reaction mixture (20 µL total volume) typically included RNA template, oligo(dT) primers, reverse transcriptase, dNTPs, and reaction buffer.
3. Reverse transcription was performed under the following conditions: 42 °C for 30 minutes, followed by 85 °C for 5 minutes to inactivate the enzyme.

Quantitative PCR (qPCR):

1. qPCR reactions were performed in 96-well plates using SYBR Green Master Mix on a Roche LightCycler 480 system (Roche, Basel, Switzerland).
2. Each 20 µL reaction contained:
   - 1. 2 µL of cDNA template
     2. 0.5 µL of forward primer (10 µM)
     3. 0.5 µL of reverse primer (10 µM)
     4. 10 µL SYBR Green Master Mix
     5. 7 µL nuclease-free water
3. Thermal cycling conditions were:
   - 1. Initial denaturation at 95 °C for 10 minutes
     2. 40 cycles of:
        - Denaturation at 95 °C for 15 seconds
        - Annealing/extension at 60 °C for 1 minute
4. Melting curve analysis was performed from 65–95 °C to verify amplification specificity.

Data analysis:

1. Relative gene expression was calculated using the comparative Ct (2^−ΔΔCt) method, normalized to β-actin as the internal control.
2. All reactions were performed in technical triplicates, and representative data were presented as mean ± standard deviation (SD).

Primer information:

1. Primer sequences for target and reference genes are listed in Table S2. Primers were designed to span exon-exon junctions when possible to avoid amplification of genomic DNA.

Critical considerations:

1. Maintain RNA integrity by using RNase-free reagents and pipette tips.
2. Avoid repeated freeze-thaw cycles of RNA and cDNA.
3. Include a no-template control (NTC) to monitor contamination.

***22. Co-immunoprecipitation (Co-IP)***

Purpose: To investigate the physical interaction between GLP-1R and AMPK in bone marrow-derived macrophages (BMDMs) under experimental conditions.

Cell lysis:

1. BMDMs were harvested and washed twice with ice-cold PBS to remove residual culture medium.
2. Cells were lysed in IP lysis buffer (20 mM Tris-HCl, pH 7.5; 150 mM NaCl; 1% NP-40; 1 mM EDTA; 10% glycerol) supplemented with protease and phosphatase inhibitor cocktail (KeyGEN, Nanjing, China) to prevent protein degradation and dephosphorylation.
3. Lysates were incubated on ice for 30 minutes with intermittent gentle vortexing every 5 minutes to ensure complete lysis.

Clarification and protein quantification:

1. Lysates were centrifuged at 12,000 × g for 15 minutes at 4 °C to remove cell debris.
2. The supernatant containing soluble proteins was carefully collected and transferred to a fresh, pre-chilled microcentrifuge tube.
3. Protein concentration was determined using a bicinchoninic acid (BCA) assay according to the manufacturer’s instructions, ensuring equal protein input for immunoprecipitation.

Pre-clearance (optional but recommended):

1. To reduce non-specific binding, lysates were pre-incubated with 30 µL of Protein A/G agarose beads for 1 hour at 4 °C with gentle rotation, followed by centrifugation to remove bead-bound proteins.

Immunoprecipitation:

1. 500 µg of total protein from each sample was incubated overnight at 4 °C with 2 µg of primary antibody: either anti-GLP-1R (Abcam, ab39072) or anti-AMPKα (Cell Signaling Technology, #5831).
2. Normal rabbit IgG (Cell Signaling Technology, #2729) was used as a negative control to assess non-specific binding.
3. Incubation was performed on a rotator to allow sufficient antibody-antigen binding.

Capture of immune complexes:

1. 30 µL of Protein A/G agarose beads (MedChemExpress, USA) were added to each tube and incubated for 2 hours at 4 °C with gentle rotation to capture the antibody-protein complexes.

Washing steps:

1. Beads were collected by brief centrifugation at 1,000 × g for 1 minute at 4 °C.
2. Supernatants were carefully discarded, and beads were washed five times with 1 mL of cold IP lysis buffer to remove non-specifically bound proteins.
3. For each wash, beads were resuspended by gentle pipetting or rotation for 5 minutes at 4 °C before centrifugation.

Elution of immunoprecipitated proteins:

1. Bound proteins were eluted by adding 30 µL of 2× SDS loading buffer and boiling the beads at 95 °C for 10 minutes to denature proteins and release them from the beads.
2. Samples were briefly centrifuged, and the supernatant containing the eluted proteins was collected for SDS-PAGE.

Western blot analysis:

1. Eluted proteins were separated by SDS-PAGE according to molecular weight and transferred to polyvinylidene fluoride (PVDF) membranes.
2. Membranes were blocked with 5% non-fat milk for 1 hour at room temperature, incubated overnight at 4 °C with primary antibodies against the target protein, followed by incubation with HRP-conjugated secondary antibodies.
3. Protein bands were visualized using a chemiluminescent imaging system (Syngene, Cambridge, UK) and analyzed with ImageJ software.

Critical considerations:

1. Keep all buffers and samples on ice to maintain protein integrity.
2. Avoid vigorous vortexing to prevent protein denaturation.
3. Include appropriate negative controls (IgG) to confirm specificity of the interaction.
4. Ensure sufficient wash steps to minimize background signal while retaining target complexes.

***23. Molecular Docking***

Purpose: To predict and analyze the potential physical interaction and binding mode between GLP-1R and AMPK at the molecular level.

Protein structure preparation:

1. The 3D structures of GLP-1R and AMPK were obtained from the Protein Data Bank (PDB) with IDs 7DUQ and 6E4U, respectively.
2. In addition, AlphaFold3 was used to predict full-length structures when high-resolution crystal structures were incomplete or missing terminal regions.
3. Low-confidence regions, such as flexible N- or C-terminal tails with predicted Local Distance Difference Test (pLDDT) < 50, were removed to improve docking accuracy.
4. Hydrogen atoms were added, and missing side chains or loops were modeled using the PyMOL (version 3.0.3) builder tool to generate complete and chemically accurate structures.

Protein preparation for docking:

1. All water molecules, ions, and co-crystallized ligands were removed from the PDB structures.
2. The protonation state of residues was adjusted according to physiological pH (~7.4).
3. The structures were energy-minimized using GROMACS 2022.3 with the AMBER99SB-ILDN force field to relieve steric clashes and optimize side-chain conformations.

Protein-protein docking:

1. Protein-protein docking was performed using the HDOCK server (http://hdock.phys.hust.edu.cn/), which implements a hybrid docking strategy combining template-based modeling and ab initio free docking.
2. The prepared GLP-1R structure was set as the receptor, and AMPK as the ligand.
3. Default parameters were used for global docking, generating top 10 potential complex models ranked by docking scores.
4. Docking was performed under rigid-body approximation, followed by semi-flexible refinement of interface residues.

Analysis and visualization:

1. Docking results were visualized and analyzed using PyMOL (version 3.0.3) to inspect interaction interfaces, hydrogen bonds, salt bridges, and hydrophobic contacts.
2. The docking score provided by HDOCK, based on knowledge-based iterative scoring functions (ITScorePP or ITScorePR), was used to evaluate binding affinity.
3. More negative docking scores indicate higher predicted binding affinity and greater confidence in the model.
4. The best-scoring docking model was further analyzed for key interface residues, potential hydrogen bonds, hydrophobic patches, and electrostatic complementarity to support subsequent mechanistic interpretation.

Critical considerations:

1. Only high-confidence, energy-minimized structures were used to avoid artifacts due to missing residues or loops.
2. Multiple docking models were compared, and consensus interfaces were considered for biological relevance.
3. Protein flexibility, solvent effects, and post-translational modifications were not fully considered in this docking; these limitations are acknowledged in the discussion.

***24. Molecular Dynamics Simulation of the GLP-1R–AMPK Complex***

Purpose: To investigate the structural stability, conformational dynamics, and interaction characteristics of the GLP-1R–AMPK complex at atomic resolution over time.

System preparation:

1. The best-scoring docking model of the GLP-1R–AMPK complex obtained from HDOCK docking was selected as the starting structure.
2. Missing hydrogen atoms were added, and the protonation state of ionizable residues was assigned according to physiological pH (~7.4) using LEaP module of AMBER24.
3. The ff19SB force field was applied to all protein residues to model bonded and non-bonded interactions accurately.
4. The system was solvated in an explicit water environment using the Optimal Point Charge (OPC) water model, in a cubic box with a 10 Å buffer around the protein complex to avoid interactions with periodic images.
5. Appropriate counterions (Na⁺ or Cl⁻) were added to neutralize the system’s net charge.

Energy minimization:

1. A two-stage energy minimization was performed to remove steric clashes and relax unfavorable contacts:
2. Initial minimization with positional restraints (10 kcal·mol⁻¹·Å⁻²) on heavy atoms for 5000 steps using steepest descent, followed by 5000 steps of conjugate gradient.
3. Unrestrained minimization for 10,000 steps to fully relax the system.

Equilibration:

1. NVT (constant number of particles, volume, and temperature) equilibration: 200 ps at 300 K using the velocity-rescale thermostat, with positional restraints on the protein backbone.
2. NPT (constant number of particles, pressure, and temperature) equilibration: 100 ps at 1 bar using the Parrinello–Rahman barostat, allowing the system density to stabilize.
3. Electrostatic interactions were treated using the particle mesh Ewald (PME) method with a 1.0 nm cutoff for nonbonded interactions, while van der Waals interactions were truncated at 1.0 nm.

Production MD simulation:

1. A 100 ns unrestrained production run was performed under NPT conditions at 300 K and 1 bar.
2. The integration time step was 2 fs, with bond lengths involving hydrogen atoms constrained using the SHAKE algorithm.
3. Trajectory coordinates were saved every 10 ps for subsequent analysis.

Trajectory analysis:

1. Post-simulation analyses were performed using Cpptraj module of AMBER24 and custom Python scripts.
2. Analyses included:
   - 1. Root-mean-square deviation (RMSD): to monitor overall structural stability of the complex over time.
     2. Root-mean-square fluctuation (RMSF): to evaluate flexibility of individual residues.
     3. Radius of gyration (Rg): to assess compactness of the protein complex.
     4. Solvent-accessible surface area (SASA): to determine solvent exposure and hydrophobic/hydrophilic surface changes.
     5. Hydrogen bond analysis: number and persistence of hydrogen bonds at the GLP-1R–AMPK interface.
3. Representative snapshots were extracted at key time points for visualization and interpretation using PyMOL 3.0.3.

Critical considerations:

1. The simulation was performed under explicit solvent and periodic boundary conditions to mimic a physiological environment.
2. Limitations include the absence of post-translational modifications and membrane environment for GLP-1R; these factors are acknowledged in the discussion.
3. All parameters were chosen to balance computational efficiency and physical realism, following best practices for protein–protein MD simulations.

***25. Behavioral Evaluation***

Purpose: To comprehensively assess hindlimb motor function and gait recovery in mice following spinal cord injury (SCI).

Basso Mouse Scale (BMS) locomotor assessment:

1. Hindlimb locomotor function was evaluated using the Basso Mouse Scale (BMS), a standardized 9-point scale ranging from 0 (complete hindlimb paralysis) to 9 (normal locomotion) .
2. Mice were acclimated to the testing environment for at least 30 minutes prior to evaluation to reduce stress-induced variability.
3. Mice were placed individually in an open field arena (50 × 50 × 20 cm) with a non-reflective floor to facilitate observation.
4. Two experienced, blinded observers independently scored hindlimb function by evaluating parameters including:
   - 1. Joint movement: ability to flex and extend hip, knee, and ankle joints.
     2. Weight support and stepping: capability to support body weight and perform plantar steps.
     3. Coordination: forelimb-hindlimb coordination during locomotion.
     4. Paw placement: proper orientation of hindpaws during stepping.
5. Each mouse was observed for 4–5 minutes, and an average score from both observers was recorded.
6. Assessments were performed pre-injury (baseline) and on days 1, 3, 7, 14, 21, and 28 post-SCI to monitor functional recovery over time.

Footprint (gait) analysis:

1. To evaluate fine motor coordination and gait parameters, footprint analysis was conducted.
2. Non-toxic red ink was applied to the forepaws and blue ink to the hindpaws of each mouse.
3. Mice were then allowed to walk along a narrow, enclosed runway (50 cm length, 5 cm width) lined with white paper to record footprints.
4. Mice were trained for 1–2 trials prior to recording to ensure consistent walking behavior.
5. Footprint patterns were analyzed to measure:
   - 1. Stride length: distance between successive placements of the same paw.
     2. Stride width: lateral distance between left and right hindpaws.
     3. Interlimb coordination: consistency of paw placement and regularity of step patterns.
6. At least 3 consecutive footprints per paw were measured and averaged for each mouse.
7. Data analysis was performed by two blinded observers to reduce scoring bias.

Additional considerations:

1. All behavioral tests were performed at the same time of day to minimize circadian variability.
2. Mice were kept on a stable temperature and humidity-controlled environment prior to testing.
3. Observers were blinded to experimental groups to ensure objective assessment.
4. Any mice that exhibited non-compliant walking behavior (e.g., stopping, jumping) were re-tested after a brief rest period.

***26. Statistical Analysis***

1. All experimental data are presented as mean ± standard error of the mean (SEM). Each experiment was independently repeated at least three times to ensure reproducibility and reliability.
2. For comparisons involving more than two groups, data were analyzed using one-way analysis of variance (ANOVA) to determine overall differences among groups. When the ANOVA indicated statistical significance, Tukey’s post hoc multiple comparisons test was applied to identify specific group differences.
3. For comparisons between two groups only, unpaired two-tailed Student’s t-tests were performed, assuming equal variance unless otherwise indicated.
4. Normality of data distribution was assessed using the Shapiro-Wilk test, and homogeneity of variance was checked using Levene’s test. If data violated assumptions of normality or equal variance, appropriate non-parametric tests (e.g., Mann–Whitney U test or Kruskal–Wallis test with Dunn’s post hoc correction) were applied.
5. All statistical analyses were performed using GraphPad Prism 10.4 (GraphPad Software, San Diego, CA, USA).
6. P-values < 0.05 were considered statistically significant, with exact P-values reported whenever possible.
7. Data visualization included bar graphs with error bars representing SEM, scatter plots, or line graphs for time-course studies. Sample sizes (n) for each experiment are indicated in figure legends.
8. To minimize bias, all analyses were performed blinded to experimental group assignments, and data points were checked for outliers using the Grubbs’ test before final statistical testing.
